# Neutrophil-to-lymphocyte ratio for primary risk stratification in acute pancreatitis: a systematic review and meta-analysis

**DOI:** 10.3389/fmed.2025.1729339

**Published:** 2026-01-13

**Authors:** Serge Chooklin, Serhii Chuklin

**Affiliations:** Surgical Center, Saint Paraskeva Medical Center, Lviv, Ukraine

**Keywords:** acute pancreatitis, biomarkers, neutrophil-to-lymphocyte ratio, organ failure, severity

## Abstract

**Background:**

Early risk stratification in acute pancreatitis should address not only the probability of severe disease but also the timely prediction of persistent organ failure, (POF) infectious complications [including infected pancreatic necrosis (IPN)], and mortality. The neutrophil-to-lymphocyte ratio (NLR) is a low-cost, first-line biomarker that is readily obtainable within the initial 24–48 h.

**Objective:**

To evaluate the clinical utility of the NLR for predicting a severe disease course, persistent organ failure, infection, and mortality across predefined, time-specific measurement windows.

**Methods:**

We conducted a systematic review with meta-analytic synthesis of studies assessing the NLR at admission and during the first two days of hospitalization. Random-effects models were used, and bivariate approaches were applied to synthesize diagnostic accuracy.

**Results:**

Elevated admission NLR was consistently associated with subsequent severe disease and an early need for intensive care. NLR dynamics on day 1 and day 2 preserved prognostic value and improved identification of patients at risk of persistent organ failure. NLR also helped identify individuals more likely to develop infectious complications, including infected pancreatic necrosis. An admission NLR > 12 was associated with in-hospital mortality. Overall, NLR showed acceptable discriminative performance with a favorable sensitivity–specificity profile and remained clinically useful for early triage.

**Conclusions:**

The NLR is a simple, reproducible tool for early prediction of organ failure, infection, and mortality in acute pancreatitis. The use of time-specific, locally calibrated thresholds, integrated with clinical scores and routine laboratory data, is recommended to optimize patient triage, routing, and care decisions.

**Systematic review registration:**

PROSPERO (CRD420251169592).

## 1 Introduction

Acute pancreatitis (AP) remains one of the leading causes of urgent hospitalization in gastroenterology and abdominal surgery, and is accompanied by a substantial burden of complications, necessitating timely triage. Contemporary clinical guidelines underscore the critical importance of risk stratification within the first 24–48 h to determine monitoring intensity, the need for transfer to the intensive care unit (ICU), and the selection of invasive strategies for necrotizing disease (1–4). Despite long-standing experience with composite scoring systems and laboratory indicators [C-reactive protein (CRP); procalcitonin (PCT)], their prognostic utility in the initial hours is limited by the lag to peak informativeness, need for extended variable sets, and inter-center variability in discriminative performance. Against this backdrop, there is a growing demand for simple, accessible, and reproducible biomarkers for early risk stratification, aligned with contemporary severity definitions under the revised Atlanta 2012 classification (5–7).

Updated guidance from professional societies emphasizes that early risk assessment should rely on tools that can be measured repeatedly without delay and anchored to clearly validated endpoints such as persistent organ failure (POF), infected pancreatic necrosis (IPN), and in-hospital mortality (1, 2). This creates a mandate for first-line indices of systemic inflammation that can be deployed in the emergency department.

The biological rationale for this strategy is grounded in early sterile inflammatory events in AP, including neutrophil activation, formation of neutrophil extracellular traps (NETs), microcirculatory disturbances, and the systemic effects of cytokines. Collectively, these processes drive an imbalance between an “aggressive” innate response and stress-induced immunosuppression, a framework that underpins the interpretation of indices, such as the neutrophil-to-lymphocyte ratio (NLR) and related metrics (systemic immune-inflammation index, SII; platelet-to-lymphocyte ratio, PLR). Accumulating literature, including reviews of NET cell biology and experimental studies of their pharmacologic modulation, reinforces this biological plausibility and highlights the potential of targeting NETs to mitigate inflammatory severity in AP (8–10).

At the same time, several fundamental questions remain unresolved (4, 11). First, the temporal behavior of early indicators is insufficiently characterized; the informativeness of biomarkers in AP changes materially between admission, 24 h, and 48 h. Even for widely used CRP, the discrimination of severe disease is strongly time-dependent; thus, fixed one-off thresholds that ignore trajectory are methodologically unsound (4, 12). Second, standardization of endpoints and thresholds is lacking; real-world implementation requires harmonized rule-in/rule-out cut-offs with sensitivity/specificity estimates, LR±, and AUC calculated within predefined temporal windows, accompanied by external validation. Third, etiologic stratification (e.g., biliary vs. hypertriglyceridemic) and comorbidity may modify the performance of inflammatory indices, yet available interaction data remain fragmentary. Fourth, comparisons with existing tools remain unsettled: it is unclear whether a simple first-line index improves the discrimination of validated scores or can function as a standalone, rapid primary triage test before composite scales are calculated.

In managing complications of necrotizing disease, it is crucial to distinguish markers of infection risk from indicators of overall severity. For infected pancreatic necrosis, the strongest evidence to date supports procalcitonin (PCT), particularly its dynamic changes, whereas C-reactive protein (CRP) more reliably reflects systemic inflammatory activity. Combining markers or using composite models may improve decision-making accuracy during critical time windows (13, 14). In parallel, emerging artificial intelligence algorithms for early severity prediction underscore the need for simple, yet informative variables available at admission (15).

In summary, current clinical guidelines provide clear management frameworks for AP while also highlighting an unmet need for rapid, standardized predictors to support early triage. Against the backdrop of the immunobiology of systemic inflammation and the practical constraints of the first hours of presentation, the NLR remains an attractive, readily measurable first-line index. Nevertheless, confident implementation will require concordant evidence regarding (i) associations with clinically meaningful outcomes within predefined temporal windows; (ii) test accuracy and operational thresholds; (iii) etiologic modification of effects; and (iv) the incremental value achieved when combined with scoring systems and/or other biomarkers.

**Study objective:** To systematically evaluate the role of the neutrophil-to-lymphocyte ratio in the early prediction of clinically meaningful outcomes in acute pancreatitis.

## 2 Methods

### 2.1 Search strategy

We searched PubMed, Scopus, and Google Scholar for studies on NLR in AP through September 2025. The search combined the keywords “neutrophil-to-lymphocyte ratio,” “NLR,” and “acute pancreatitis.” In parallel, two reviewers independently screened the reference lists of included articles to identify additional publications and maximize completeness. Disagreements regarding study relevance were resolved through discussion until consensus was reached. The protocol for this systematic review and meta-analysis was registered in PROSPERO (CRD420251169592).

### 2.2 Review design and questions

We conducted a prespecified systematic review comprising two complementary syntheses:

- Meta-analysis of prognostic factors: assessment of the association between NLR and adverse AP outcomes using odds ratios (ORs) or relative risks (RRs).
- Meta-analysis of prognostic accuracy: pooling the discriminatory performance of NLR [area under the receiver operating characteristic curve (AUC), sensitivity/specificity, positive/negative likelihood ratios (LR+/LR–), and diagnostic odds ratio (DOR)] based on 2 × 2 tables (TP/FP/FN/TN) and reported AUC values.

Reporting adhered to PRISMA and MOOSE for meta-analyses of observational studies, and PRISMA-DTA for reviews of diagnostic accuracy. The clinical questions were as follows: (1) Is a higher NLR associated with severe disease, persistent organ failure (POF), infected pancreatic necrosis (IPN), or in-hospital mortality? (2) How accurately does NLR measured at different time points (day 0, day 1, and day 2) discriminate these outcomes?

### 2.3 Inclusion and exclusion criteria

Eligible studies were original investigations of adult patients with AP in which NLR was measured during the index hospitalization or within the first 48 h (strata: day 0, ~24 h/day 1, ~48 h/day 2) and were related to at least one of the following outcomes: severe disease (SAP/severe AP), POF, IPN, or in-hospital mortality.

For the prognostic factor synthesis, studies were required to report odds ratios (ORs) or risk ratios (RRs), adjusted and/or unadjusted, or to provide sufficient data for their calculation. For the prognostic accuracy synthesis, studies had to report 2 × 2 tables and/or the area under the curve (AUC) with 95% confidence intervals (CIs). We excluded pediatric cohorts, single-case reports, narrative reviews, and publications lacking data suitable for quantitative synthesis.

### 2.4 Endpoint definitions and time windows

The primary outcomes were SAP, defined according to the revised Atlanta classification or accepted equivalents; POF, as defined by the source studies; IPN; and in-hospital mortality. Analytic time windows were harmonized into three strata: admission (day 0), day 1 (~24 h), and day 2 (~48 h).

### 2.5 Data collection and risk of bias assessment

Two reviewers independently screened titles/abstracts and full texts and extracted data using a standardized form (study identifier; design; sample size; timing and method of NLR measurement; NLR threshold; number of events; 2 × 2 contingency tables; AUC with 95% CI; OR/RR with 95% CI; and covariates included in adjusted models). For reporting of the prognostic meta-analysis, we used the MOOSE checklist (16), and for diagnostic sub-analyses we followed PRISMA-DTA (17). Discrepancies during study selection and data extraction were resolved by consensus, with involvement of a third reviewer when needed.

Risk of bias for prognostic factor studies was assessed using the QUIPS tool (six domains: study participation, study attrition, prognostic factor measurement, outcome measurement, confounding, and statistical analysis/reporting) (18). For prognostic accuracy sub-analyses, we used QUADAS-2 (19). Domain-level judgments were incorporated into sensitivity analyses. The conduct and reporting of this study adhered to contemporary principles of transparency and reproducibility; in particular, recent recommendations such as TITAN for studies involving artificial intelligence were considered (20).

### 2.6 Statistical analysis

#### 2.6.1 Prognostic factor (OR/RR associations)

Effect estimates (OR/RR) were synthesized on a log scale using random-effects models (REML) with Hartung–Knapp–Sidik–Jonkman confidence intervals. Heterogeneity was described using *I*^2^ and τ^2^, and PIs were reported when feasible. When multiple estimates were available within a study, we prioritized pre-specified early time windows (admission/day 1/day 2) and adjusted the models when reported.

Given the anticipated clinical and methodological diversity in acute pancreatitis (etiology, baseline risk, timing of blood sampling, and outcome definitions), substantial heterogeneity was expected. Accordingly, we interpreted *I*^2^ alongside τ^2^ and 95% prediction intervals and focused on clinically interpretable accuracy measures (HSROC-derived Se/Sp and LR±) with locally calibrated thresholds rather than on a single universal pooled cut-off.

#### 2.6.2 Prognostic accuracy (DTA approach)

For studies providing complete 2 × 2 data, we calculated sensitivity and specificity and estimated pooled accuracy using a bivariate HSROC (Reitsma) model. Where available, AUC values were pooled using random-effects inverse-variance methods. Small-study effects in diagnostic accuracy analyses were explored using Deeks' test; additional model diagnostics are provided in the Supplementary Materials.

#### 2.6.3 Thresholds and clinical calibration

Reported NLR cut-offs were summarized according to outcome and time window. Working thresholds were proposed by integrating the HSROC summary point with the empirical distribution of published cut-offs and were illustrated with post-test probabilities under clinically plausible prevalence (details in Supplementary Tables and Figures).

#### 2.6.4 Subgroup and sensitivity analyses

Pre-specified subgroup analyses (e.g., etiology and sampling time) and sensitivity analyses (e.g., influence/leave-one-out checks and alternative assumptions for sparse data) were performed when sufficient studies were available; complete outputs are reported in the Supplementary materials.

#### 2.6.5 Software and reproducibility

All analyses were performed in R (version 4.5.1); the full analytic workflow and technical specifications are detailed in the Supplementary materials. As an auxiliary check for DOR coherence and rapid SROC construction in the selected sub-analyses, we used Meta-DiSc 2.0.

## 3 Results

**Figure 1:**
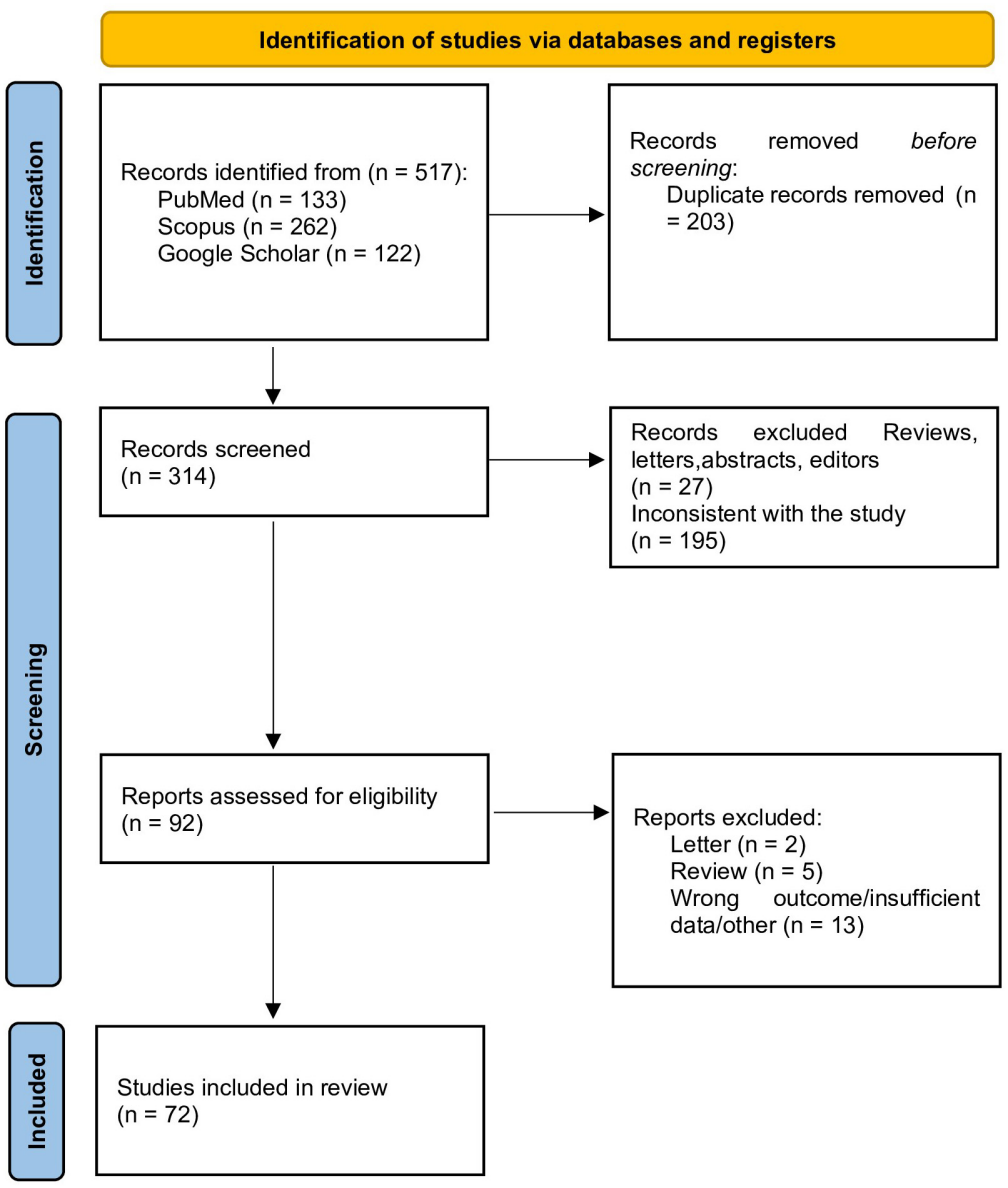
PRISMA 2020 flow diagram for study selection.

In accordance with PRISMA 2020 recommendations (21), 517 records were screened across databases; 92 reports underwent full-text assessment, and 72 studies (6, 22–92) were included in the qualitative synthesis (Table 1 and Figure 1). Risk of bias was evaluated using QUIPS (18) (Figures S1, S2). Overall, studies were rated as having low-to-moderate risk of bias, and none were rated as high risk.

**Table 1:** Characteristics of studies of the neutrophil-to-lymphocyte ratio in acute pancreatitis.

**Study**	**Year (patient accrual)**	**Country**	**Study design**	**Number of patients**	**Etiology**	**Severity assessment**	**Timing of NLR measurement**	**Prediction (endpoints)**
Vo et al. (22)	2023 (2021–2022)	Vietnam	Prospective	131	AP, BP, HTG, AP+HTG, other	Atlanta 2012	On admission	1
Lu et al. (23)	2022 (2016–2019)	China	Retrospective	446	HTG	Atlanta 2012, Ranson, BISAP, APACHE II	On admission	1, 2
Huang et al. (24)	2019 (2012–2017)	China	Retrospective	268	AP, BP, HTG	Atlanta 2012	On admission	1
Halaseh et al. (25)	2022 (2019–2020)	United Kingdom	Retrospective	314	Mixed cohort	Glasgow	On admission; at 24 and 48 h	4, 5
Abayli et al. (26)	2018	Turkey	Retrospective	435	Mixed cohort	Ranson	On admission	1
Kolber et al. (27)	2018	Poland	Prospective	95	Mixed cohort	Atlanta 2012	On admission; at 24 and 48 h	2
Park et al. (28)	2019 (2008–2017)	Republic of Korea	Retrospective	672	Mixed cohort	Atlanta 2012	On admission	1
Petrescu et al. (29)	2019 (2013–2017)	Romania	Retrospective	337	Mixed cohort	Balthazar	On admission	1
Tahir et al. (30)	2021 (2016–2019)	Pakistan	Retrospective	166	Mixed cohort	Atlanta 2012, MCTSI	On admission	1
Xu et al. (31)	2024 (2012–2023)	China	Retrospective	771	BP, HTG	Atlanta 2012, BISAP, MCTSI	On admission	2
Karabuga et al. (32)	2022 (2019–2020)	Turkey	Retrospective	500	BP, other	BISAP	On admission	1
Wang et al. (33)	2017 (2010–2016)	USA	Retrospective	110	HTG	Atlanta 2012, Ranson, APACHE II	On admission	1, 4
Cho et al. (34)	2018 (2014–2016)	Republic of Korea	prospective	243	AP, BP	Atlanta 2012, Ranson, BISAP, CTSI	On admission	2
Zhou et al. (35)	2019 (2014–2017)	China	Retrospective	406	AP, BP, HTG, other	Atlanta 2012, BISAP, Ranson, APACHE II	On admission	1, 4
Azab et al. (36)	2011 (2005–2008)	USA	Retrospective	283	Mixed cohort	Modified Early Warning Score (MEWS)	On admission	1, 4
Gezer et al. (37)	2020 (2015–2018)	Turkey	Retrospective	80	Mixed cohort	Atlanta 2012, HAPS, BISAP, Ranson, MCTSI, Balthazar	On admission	1, 4
Yu et al. (38)	2019 (2013–2018)	China	Retrospective	159	HTG, other	Atlanta 2012	On admission	1
Suppiah et al. (39)	2013 (2010)	United Kingdom	Retrospective	146	Mixed cohort	Atlanta 2012, Glasgow	On admission; at 24 and 48 h	1, 2, 5
Reddy and Muduru (40)	2025 (2022–2024)	India	Prospective	70	Mixed cohort	Atlanta 2012	On admission; at 24, 48 and 72 h	1, 2, 4
O'Connell et al. (41)	2018 (2013–2016)	Ireland	Retrospective	185	Mixed cohort	ICU/HDU	On admission	1, 4
Vincent and Shashirekha (42)	2024	India	Retrospective	118	Mixed cohort	Atlanta 2012, CTSI	On admission	1
Jeon and Park (43)	2017 (2007–2017)	Republic of Korea	Retrospective	490	Mixed cohort	Atlanta 2012	On admission; at 24, 48 and 72 h	1, 2
Ak et al. (44)	2023 (2015–2020)	Turkey	Retrospective	514	Mixed cohort	Atlanta 2012	On admission	1, 4
Han et al. (45)	2017	China	Retrospective	328	BP, other	Atlanta 2012, Ranson	On admission; at 24 and 48 h	1
Kumbhar et al. (46)	2025 (2018–2022)	India	Retrospective	505	Mixed cohort	Atlanta 2012, BISAP	At onset of fever	3
Uludag et al. (47)	2022 (2012–2018)	Turkey	Retrospective	341	Mixed cohort	Balthazar CTSI	On admission	1
Jain et al. (48)	2023 (2020–2022)	India	Prospective	249	Mixed cohort	Atlanta 2012, APACHE II, SAPS II, BISAP	On admission	4
Lu et al. (49)	2023	China	Retrospective	113	AP, BP, HTG, other	WSES-2019, BISAP	On admission	1
Akdur et al. (50)	2022	Turkey	Retrospective	171	Mixed cohort	Atlanta 2012, BISAP	On admission	1, 5
Junare et al. (51)	2021 (2018–2019)	India	Prospective	160	Mixed cohort	Atlanta 2012, APACHE II, BISAP, mCTSI	On admission	2, 4
Li et al. (52)	2017 (2013–2015)	China	Retrospective	359	AP, BP, HTG, other	Atlanta 2012	On admission	1, 4
Dancu et al. (53)	2021 (2018–2019)	Romania	Retrospective	216	Mixed cohort	Atlanta 2012, BISAP	On admission; at 48 h	1, 4, 5
Sandhyav et al. (54)	2025 (2015–2018)	India	Prospective	39	Mixed cohort	Atlanta 2012, CTSI	At signs of infection	3
Pian et al. (55)	2021 (2015–2017)	China	Retrospective	169	AP, BP, HTG, other	Atlanta 2012	On admission	1
Liu et al. (56)	2022 (2010–2020)	China	Retrospective	2327	AP, BP, HTG, other	Atlanta 2012, CTSI	On admission	1
Mihoc et al. (57)	2021 (2016–2020)	Romania	Retrospective	53	BP, HTG, other	Atlanta 2012, CTSI	On admission	4
Xinyu et al. (58)	2025 (2021–2023)	China	Retrospective	137	Mixed cohort	Atlanta 2012	On admission	1
Ünal and Barlas (59)	2019	Turkey	Retrospective	96	BP, other	mCTSI	On admission	1
Liu et al. (60)	2021 (2020–2021)	China	Retrospective	101	Mixed cohort	Atlanta 2012, BISAP, CTSI	On admission	1
Mubder et al. (61)	2020 (2015–2018)	USA	Retrospective	239	Mixed cohort	Atlanta 2012	On admission; at 24 and 48 h	1, 2, 4
Lin et al. (62)	2023 (2012–2021)	China	Retrospective	311	HTG	Atlanta 2012	On admission	1
Khan et al. (63)	2021	Pakistan	Retrospective	154	Mixed cohort	Balthazar	On admission	1
Li et al. (64)	2024 (2018–2020)	China	Retrospective	253	Mixed cohort	Atlanta 2012, BISAP, Ranson	On admission	1
Bengi et al. (65)	2025 (2021–2023)	Turkey	Retrospective	238	BP, other	BISAP, Ranson, HAPS	On admission; at 24 and 48 h	1, 2, 4
Piñerúa-Gonsálvez et al. (66)	2025 (2014–2022)	Spain	Retrospective	778	Mixed cohort	Atlanta 2012, BISAP, Ranson	On admission	2
Zengin et al. (6)	2025 (2019–2024)	Turkey	Retrospective	412	BP, other	Atlanta 2012, BISAP, Ranson, Glasgow, APACHE II	On admission	4, 5
Araiza-Rodríguez et al. (67)	2025 (2021–2023)	Mexico	Retrospective	100	BP	Atlanta 2012	On admission	1
Qi et al. (68)	2025 (2020–2023)	China	Retrospective	415	AP, BP, HTG, other	Atlanta 2012	On admission	3
Saribas et al. (69)	2025 (2017–2024)	Turkey	Retrospective	209	BP	Atlanta 2012	On admission	4
Kurtipek et al. (70)	2025 (2021–2023)	Turkey	Prospective	424	Mixed cohort	Atlanta 2012	On admission	1
Huynh et al. (71)	2025 (2022–2023)	Vietnam	Prospective	340	Mixed cohort	Atlanta 2012	On admission	1
Bhanou et al. (72)	2018	India	Retrospective	107	Mixed cohort	Atlanta 2012	On admission	1, 2, 4
Memmedova et al. (73)	2019 (2014–2016)	Turkey	Retrospective	150	BP, other	Ranson, Glasgow, Balthazar	On admission	1, 5
Fonseca et al. (74)	2021	Portugal	Retrospective	445	Mixed cohort	Atlanta 2012	On admission	1, 2, 4
Roy et al. (75)	2023	Bangladesh	Prospective	120	Mixed cohort	Atlanta 2012	On admission	1
Abu-Elfatth et al. (76)	2022 (2018–2020)	Єгипет	Prospective	100	Mixed cohort	Atlanta 2012	On admission	1, 4
Shrestha et al. (77)	2024 (2020–2021)	Nepal	Prospective	45	AP, BP, other	Atlanta 2012	On admission	1, 4
Yalçin and Yalaki (78)	2019	Turkey	Retrospective	667	BP, other	Atlanta 2012, Ranson	At 48 h	4
Kumar and Tamma (79)	2023 (2022)	India	Prospective	48	Mixed cohort	Atlanta 2012	On admission; at 24 and 48 h	1
Mumin et al. (80)	2024	Bangladesh	Prospective	40	Mixed cohort	BISAP	On admission	1, 4
Pati et al. (81)	2025 (2020–2021)	India	Prospective	108	AP, BP, HTG, other	Atlanta 2012, BISAP	On admission	1, 4
Orak et al. (82)	2016 (2009–2014)	Turkey	Retrospective	494	Mixed cohort	Atlanta 2012	On admission	1, 4, 5
Ergenc et al. (83)	2022 (2014–2015)	Turkey	Retrospective	200	BP, other	Atlanta 2012	On admission; at 48 h	1
Silva-Vaz et al. (84)	2020 (2015–2017)	Portugal	Prospective	75	BP	Atlanta 2012, BISAP	On admission	1, 4
Pan et al. (85)	2023 (2021–2023)	China	Retrospective	145	HTG	Atlanta 2012	On admission	1
Mihoc et al. (86)	2025 (2017–2024)	Romania	Retrospective	179	Mixed cohort	Atlanta 2012, APACHE II, Ranson, CTSI	On admission	4
Aktaş et al. (87)	2025 (2018–2022)	Turkey	Retrospective	357	Mixed cohort	Atlanta 2012, APACHE II, BISAP, mCTSI	On admission; at 24 and 48 h	1, 4, 5
Zhu et al. (88)	2023 (2018–2020)	China	Retrospective	324	BP, HTG, other	Atlanta 2012	On days 1, 4, and 7	3
Shabbir et al. (89)	2025 (2017–2018)	USA	Retrospective	1 250	Mixed cohort	Atlanta 2012	On admission	1
Jin et al. (90)	2021 (2017–2019)	China	Retrospective	300	BP, HTG, other	Atlanta 2012	On admission	1
Sarihan et al. (91)	2024 (2019–2022)	Turkey	Retrospective	254	Mixed cohort	MCTSI	On admission	1
Cazacu et al. (92)	2023 (2018–2021)	Romania	Retrospective	725	AP, BP, HTG, other	Atlanta 2012, BISAP, SOFA, mCTSI	On admission; at 48 h	2, 4

1 severe course, 2 organ failure, 3 infected pancreatic necrosis, 4 mortality, 5 complications, AP, alcoholic pancreatitis; BP, biliary; HTG, hypertriglyceridemic.

### 3.1 NLR on admission

**Figure 2:**
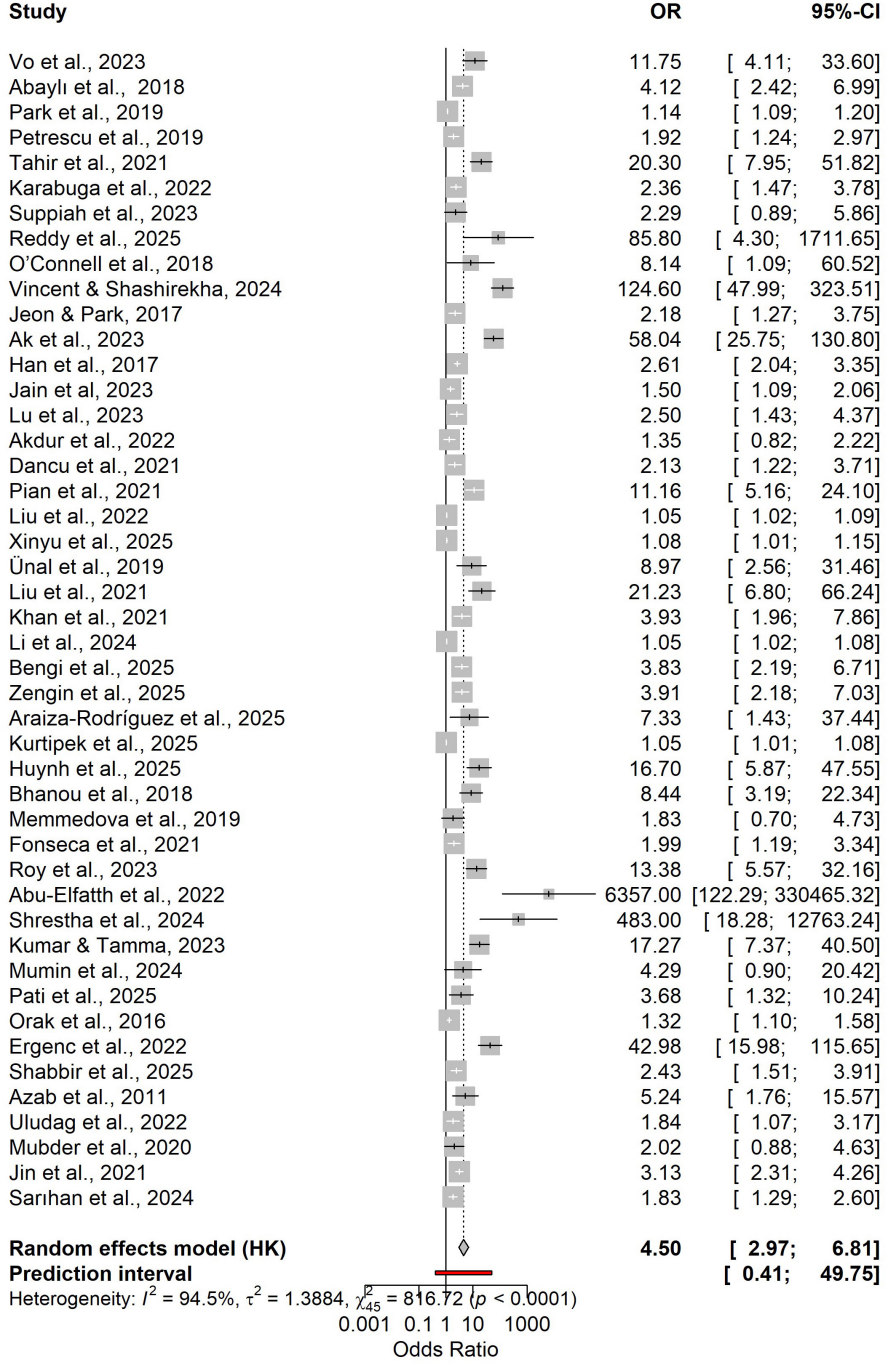
Forest plot of odds ratios (ORs) for day-0 NLR and the risk of a severe course of acute pancreatitis.

**Figure 3:**
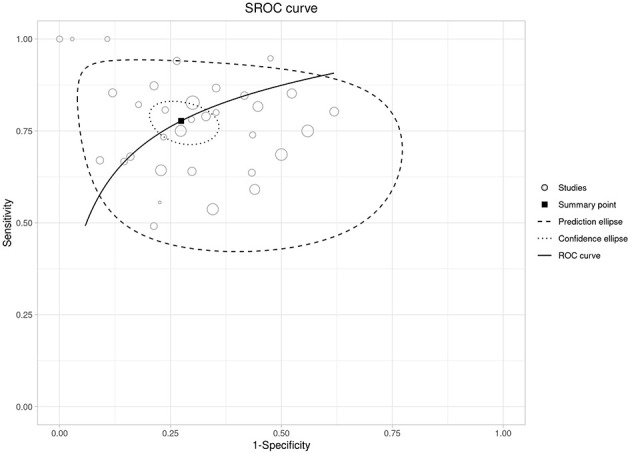
Summary ROC (SROC) curve for admission NLR with the summary point and confidence/prediction ellipses.

Higher admission NLR was associated with an increased risk of severe disease, with a pooled OR of 4.50 (95% CI 2.97–6.81) and a wide prediction interval (0.41–49.75; Figure 2 and Table S1). In the bivariate diagnostic test accuracy meta-analysis (33 studies; 7,223 patients), the summary estimates were sensitivity 0.777 and specificity 0.726, with a summary AUC of 0.771, indicating good discrimination despite substantial between-study heterogeneity (Figures 3, S3–S5). Published cut-offs clustered around 7–10 and are summarized in Table S2, while variability in log(DOR) with prediction intervals is shown in Figure S6. Based on these distributions and pooled performance, we selected an operational threshold of NLR = 9; corresponding PPV/NPV across assumed prevalences (10–30%) are presented in Figure S7 and Table S3. Threshold-category behavior on the HSROC plane is shown in Figure S8.

#### 3.1.1 NLR on day 1

**Figure 4:**
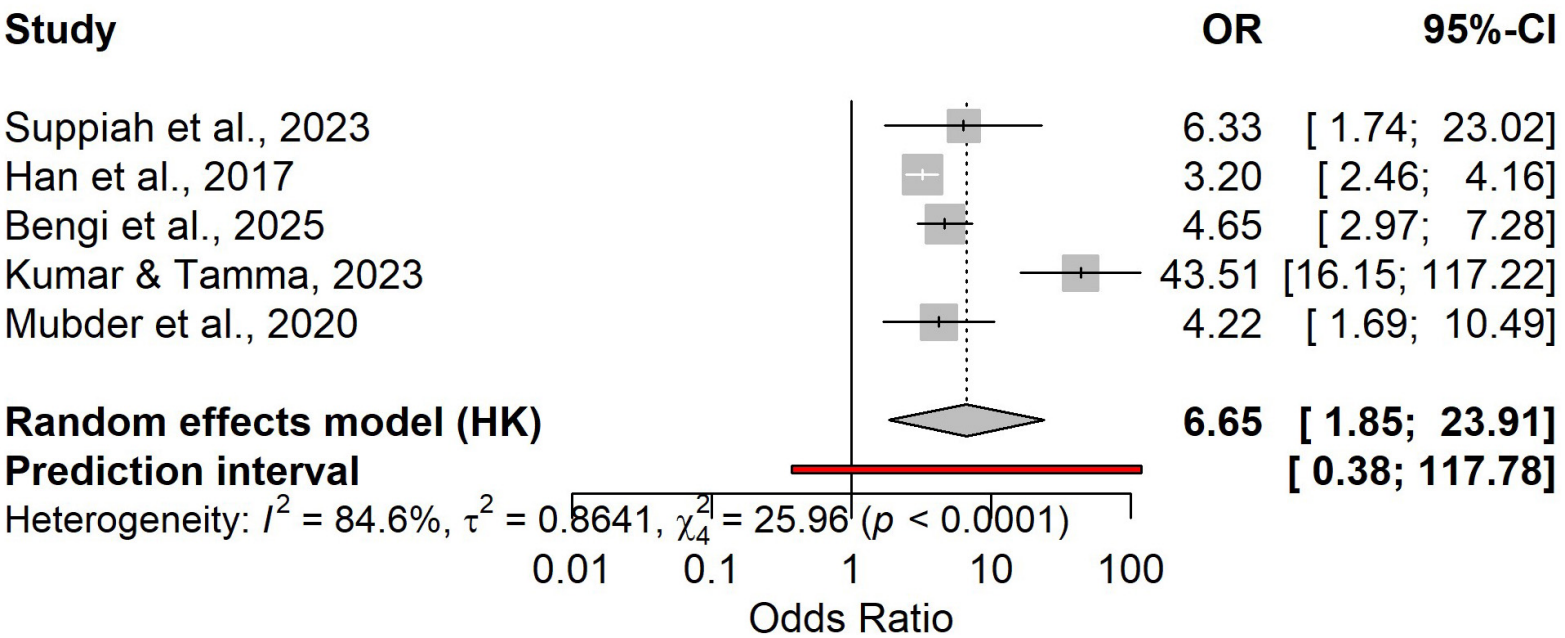
Forest plot of odds ratios (OR) for day-1 NLR and the risk of a severe course of acute pancreatitis.

**Figure 5:**
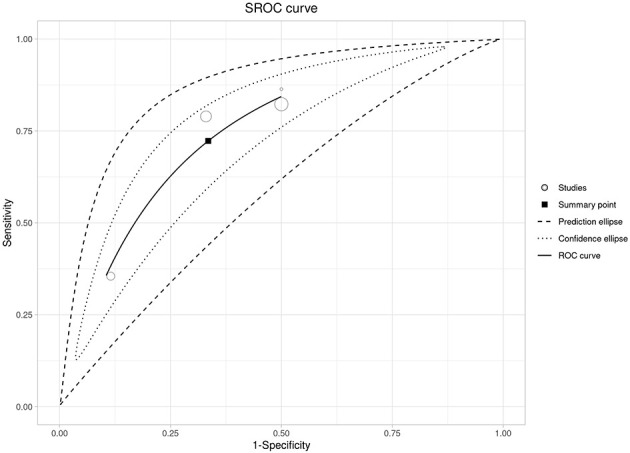
Summary ROC (SROC) curve for day-1 NLR with the summary point and confidence/prediction ellipses.

Five studies showed that higher day-1 NLR was associated with severe disease (pooled OR 6.65, 95% CI 1.85–23.91), with high heterogeneity and a wide prediction interval (0.38–117.78; Figure 4). In the bivariate diagnostic test accuracy meta-analysis (4 studies; *n* = 833; prevalence 0.30), pooled sensitivity was 0.723 and pooled specificity was 0.665, corresponding to a DOR of 5.17, LR+ of 2.16, and LR– of 0.417 (Figures S9, S10, S12). Discrimination was moderate to good (AUC 0.779) in the SROC analysis (Figures 5, S11). Reported cut-offs clustered around ~8 (weighted median 8.1; Table S4); using NLR = 8.1, expected PPV/NPV were 0.19/0.96 (10%), 0.35/0.91 (20%), and 0.48/0.85 (30%; Figure S13 and Table S5).

#### 3.1.2 NLR on day 2

**Figure 6:**
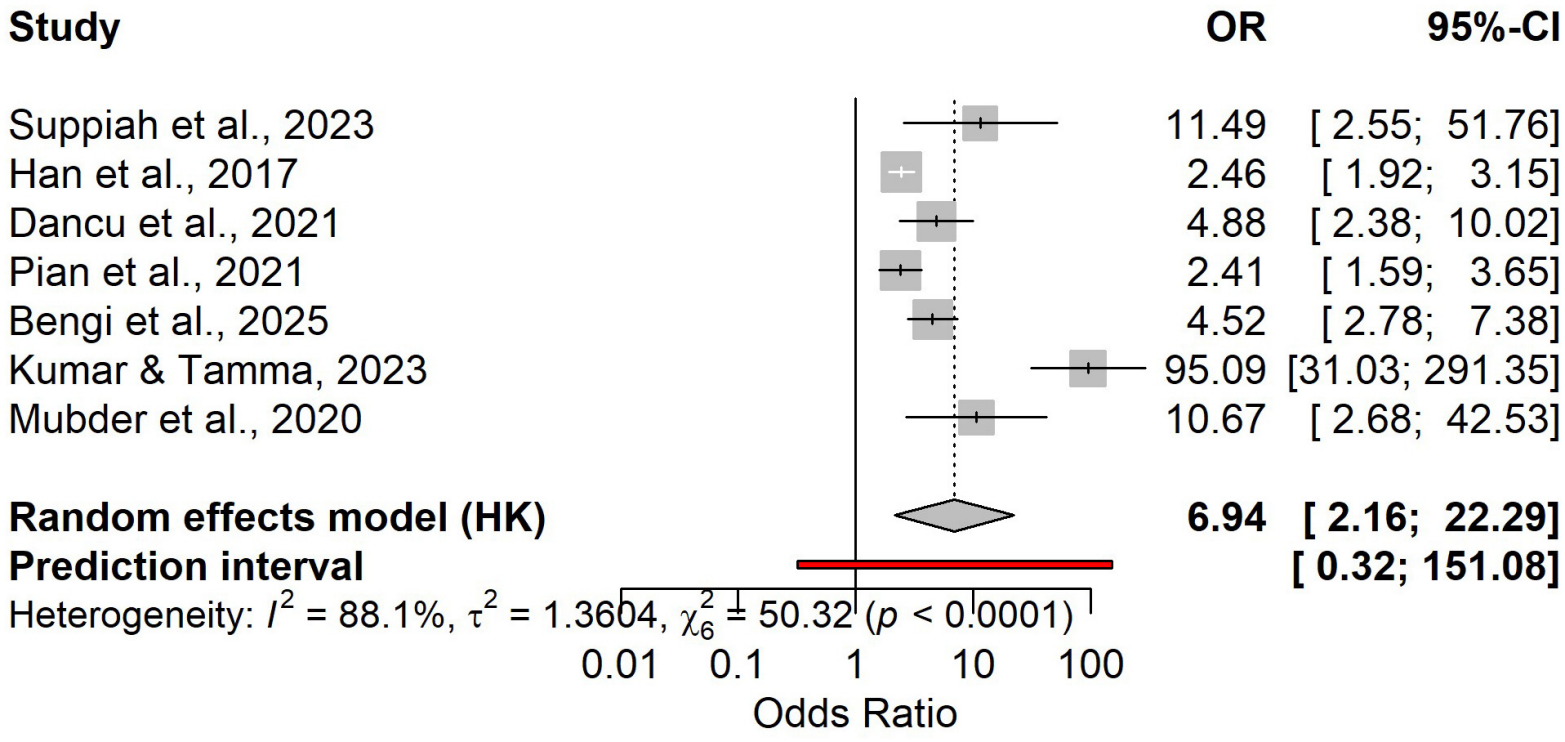
Forest plot of odds ratios (OR) for day-2 NLR and the risk of a severe course of acute pancreatitis.

**Figure 7:**
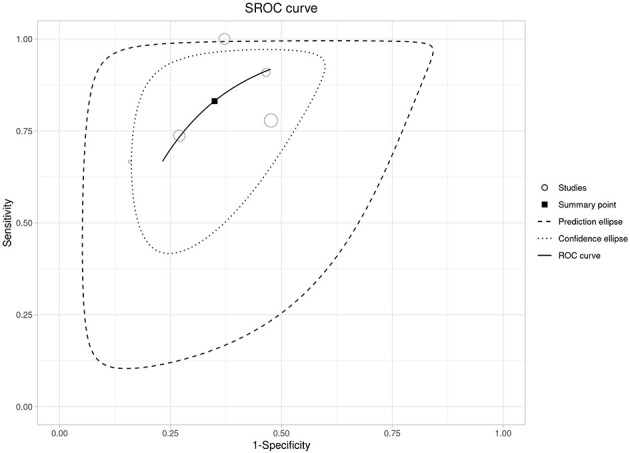
Summary ROC (SROC) curve for day-2 NLR with the summary point and confidence/prediction ellipses.

Seven studies contributed to the association analysis, showing that higher day-2 NLR was linked to severe acute pancreatitis (random-effects OR 6.94, 95% CI 2.16–22.29), with substantial heterogeneity and a wide prediction interval (0.32–151.08; Figure 6). In the bivariate diagnostic test accuracy meta-analysis (5 studies; *n* = 958; prevalence 0.27), pooled sensitivity was 0.831 (95% CI 0.674–0.921) and pooled specificity was 0.651 (95% CI 0.541–0.747; Figures S14, S15), corresponding to a DOR of 9.17 (95% CI 4.04–20.85), LR+ of 2.38, and LR– of 0.259 (Figure S18). The SROC analysis showed moderate to good discrimination (AUC 0.753, 95% CI 0.696–0.802; Figures 7, S16, S17). Reported cut-offs (*n* = 6) clustered around ~6–8 (weighted median 6.5; median 7.0; IQR 6.24–9.75; Table S6); therefore, we used an operational threshold of NLR ≈ 7 on day 2. Using LR+ 2.38 and LR– 0.259, expected PPV/NPV are 0.21/0.97 at 10% prevalence, 0.37/0.94 at 20%, 0.47/0.91 at 27%, and 0.50/0.90 at 30% (Figure S19 and Table S7).

#### 3.1.3 NLR in acute biliary pancreatitis

**Figure 8:**
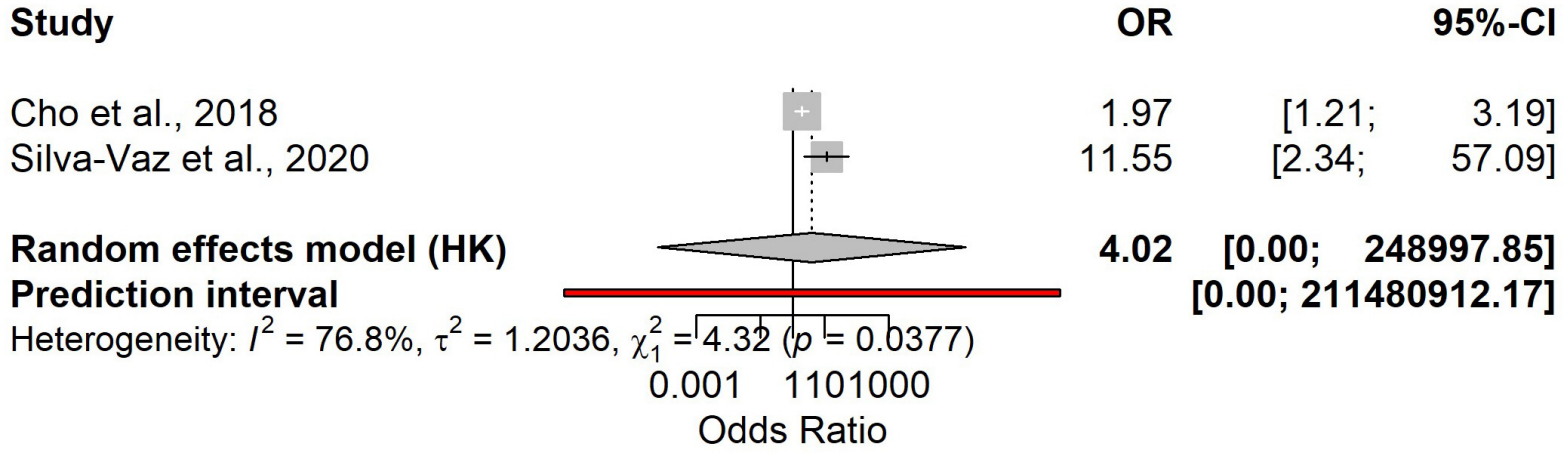
Forest plot of pooled odds ratios (ORs) for the association between elevated admission NLR and a severe course of acute biliary pancreatitis.

Two studies (*n* = 209; severe ABP = 19; prevalence 9%) evaluated admission NLR. Pooled diagnostic accuracy showed sensitivity 0.84 (95% CI 0.61–0.95) and specificity 0.54 (95% CI 0.35–0.72; Figures S20, S21), corresponding to LR+ 1.83, LR– 0.29, and DOR 6.28 (Figure S23); AUC is summarized in Figure S22. Sensitivity heterogeneity was negligible (*I*^2^ = 0%), whereas specificity was highly heterogeneous (*I*^2^ = 91%), consistent with different thresholds across studies. The association meta-analysis suggested an increased risk of severe ABP with elevated admission NLR (OR 4.02), but with extremely wide confidence intervals due to few events (Figure 8). Reported cut-offs differed (7.80 vs. 14.64); a pragmatic working threshold was NLR ≈ 10 (range 8–11). At a 9% prevalence, the expected PPV is approximately 0.15 and the NPV approximately 0.97, indicating that NLR is primarily useful for ruling out a severe course at intake.

#### 3.1.4 NLR in hypertriglyceridemia-induced pancreatitis (HTG-AP)

**Figure 9:**
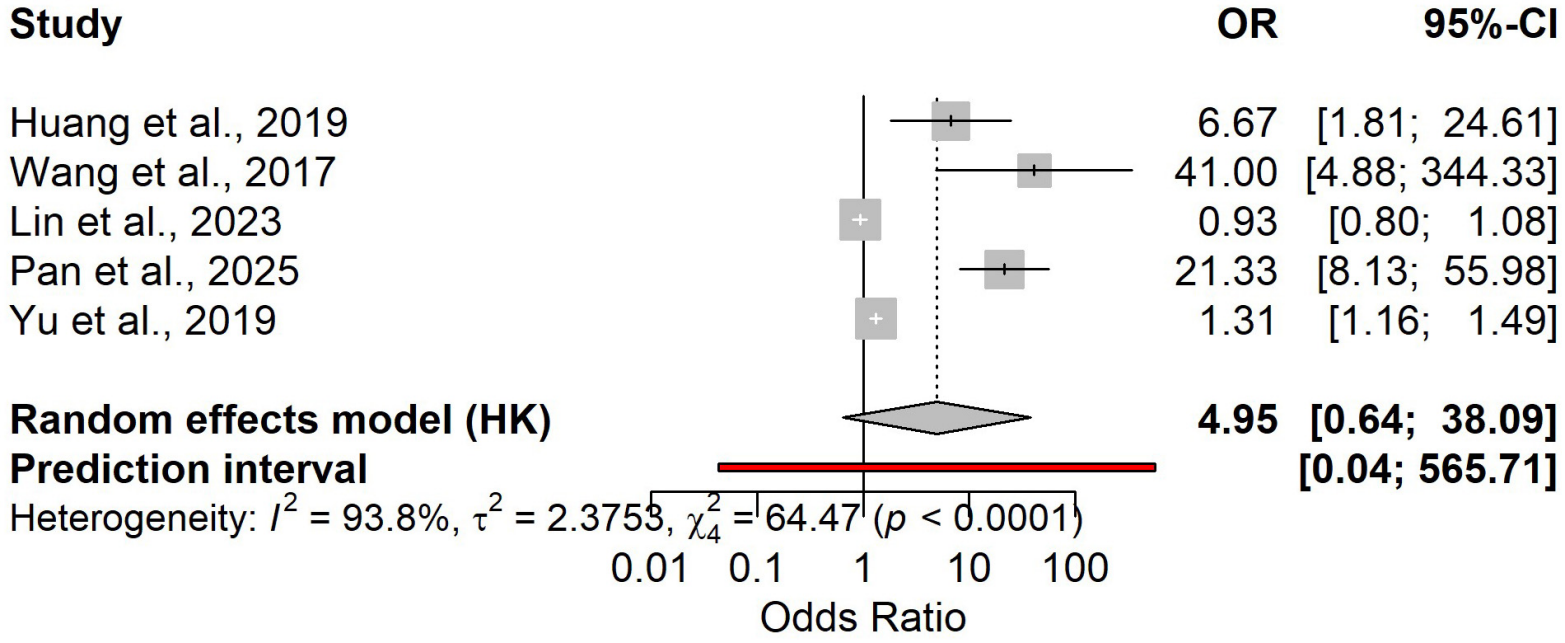
Forest plot of odds ratios (ORs) for the association between elevated admission NLR and a severe course of hypertriglyceridemia-induced acute pancreatitis (random-effects Hartung–Knapp model).

Three studies (*n* = 378; severe = 112; prevalence 30%) assessed admission NLR diagnostic accuracy: Se 0.90 (95% CI 0.83–0.94) and Sp 0.68 (95% CI 0.51–0.81; Figures S26, S27), with LR+ 2.79, LR– 0.15, DOR 19.22, and pooled AUC 0.75 (95% CI 0.646–0.834; Figures S28, S29). Sensitivity heterogeneity was minimal (*I*^2^ = 0%), whereas specificity heterogeneity was high (*I*^2^ = 90%), again suggesting threshold variability. The association meta-analysis (five studies) yielded an OR of 4.95 (95% CI 0.64–38.09) with substantial heterogeneity (*I*^2^ = 93.8%; Figure 9). Reported cut-offs ranged from 5.88 to 10.00; overall performance favored an operational threshold of NLR ≈ 10 (practical range 8–11). At a 30% prevalence, the expected PPV is approximately 0.55 and the NPV approximately 0.94.

### 3.2 NLR for organ dysfunction

**Figure 10:**
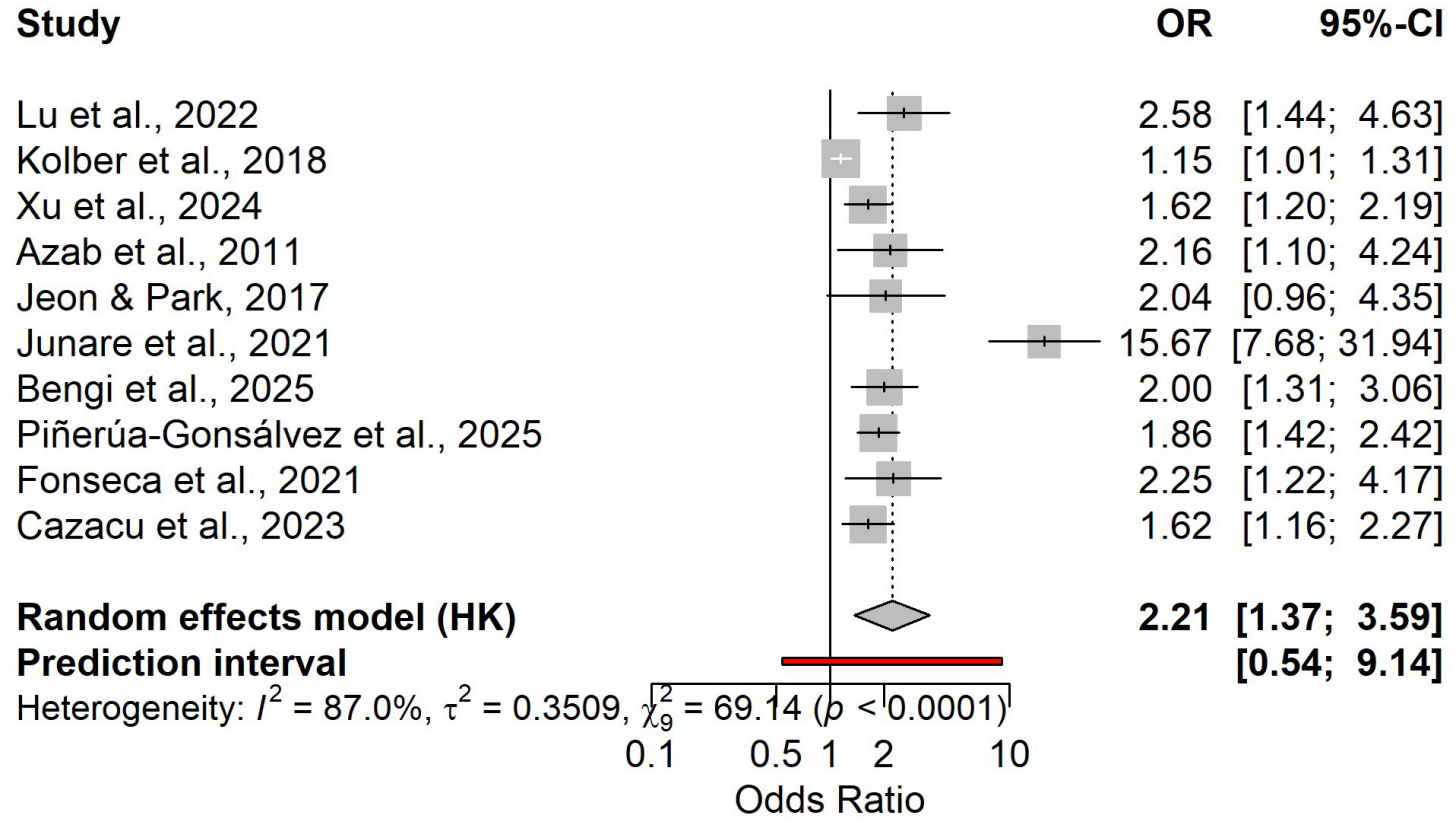
Pooled forest plot of the association between admission NLR and risk of POF (random-effects model).

**Figure 11:**
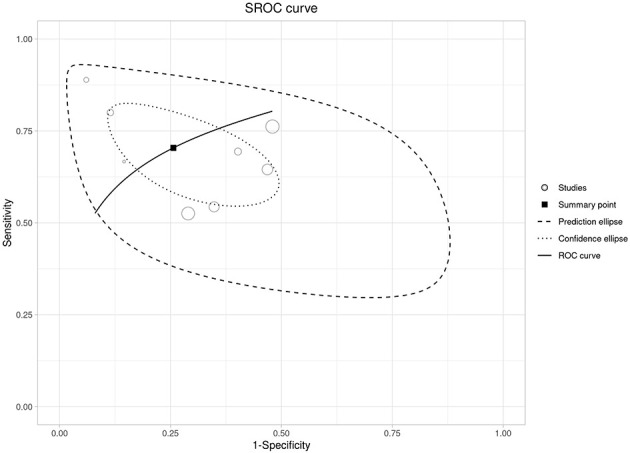
SROC curve for admission NLR (POF) with the summary point and predictive/confidence ellipses.

Ten studies assessed admission NLR as a prognostic marker for subsequent POF; all contributed to the pooled effect estimate, and eight reported 2 × 2 data suitable for diagnostic accuracy synthesis (*n* = 3,178; POF prevalence 12%). Overall, higher admission NLR was associated with increased odds of POF in the random-effects meta-analysis, with substantial between-study heterogeneity and a wide prediction interval (Figure 10). In the bivariate HSROC model (8 studies), the summary operating point indicated moderate sensitivity and specificity for identifying patients who will develop POF, consistent with the SROC curve and the dispersion of study points and confidence/prediction regions (Figures 11, S30, S31). Discrimination was modest in the pooled AUC meta-analysis, aligning with the HSROC summary performance (Figures 11, S33), while diagnostic odds ratios varied across studies with persistent heterogeneity (Figure S34). Reported “optimal” admission NLR thresholds ranged widely, with a central tendency around ~9, supporting a pragmatic cut-off near this value with local calibration to baseline risk (Table S8 and Figure S32). Using the summary likelihood ratios, post-test values at representative prevalences are summarized in Table S9 and illustrated with Fagan nomograms (Figure S35).

### 3.3 NLR for infected pancreatic necrosis (IPN)

**Figure 12:**
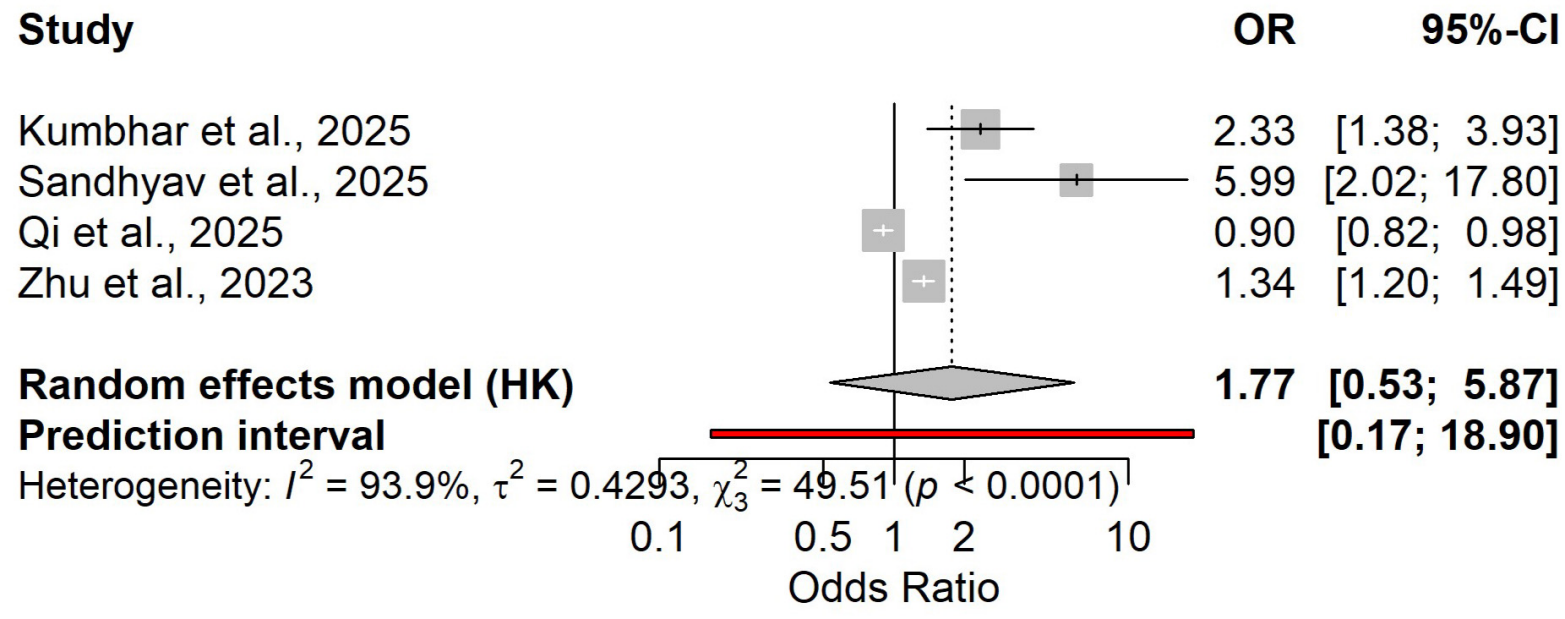
Forest plot of the association between NLR and infected pancreatic necrosis (OR, 95% CI; random-effects model).

Four studies (*n* = 744; IPN prevalence ~45%) assessed admission NLR. The pooled association with IPN was imprecise and highly heterogeneous (OR 1.77, with a wide prediction interval; Figure 12). Given the extreme between-study heterogeneity and the small number of studies, this pooled estimate should be considered exploratory and should not be used for definitive inference. In the HSROC analysis, diagnostic accuracy was moderate (Se ≈ 0.75; Sp ≈ 0.67; LR+ ≈ 2.26; LR– ≈ 0.38; DOR in the moderate range; Figures S36, S37, S39). Discrimination was generally good but variable (pooled AUC ≈ 0.79; Figure S38). Reported cut-offs (~3.5–6.2) support a pragmatic threshold around NLR ≈ 6, with local calibration at ~45% prevalence, PPV ≈ 0.65 and NPV ≈ 0.76 (illustrative).

### 3.4 NLR and mortality

**Figure 13:**
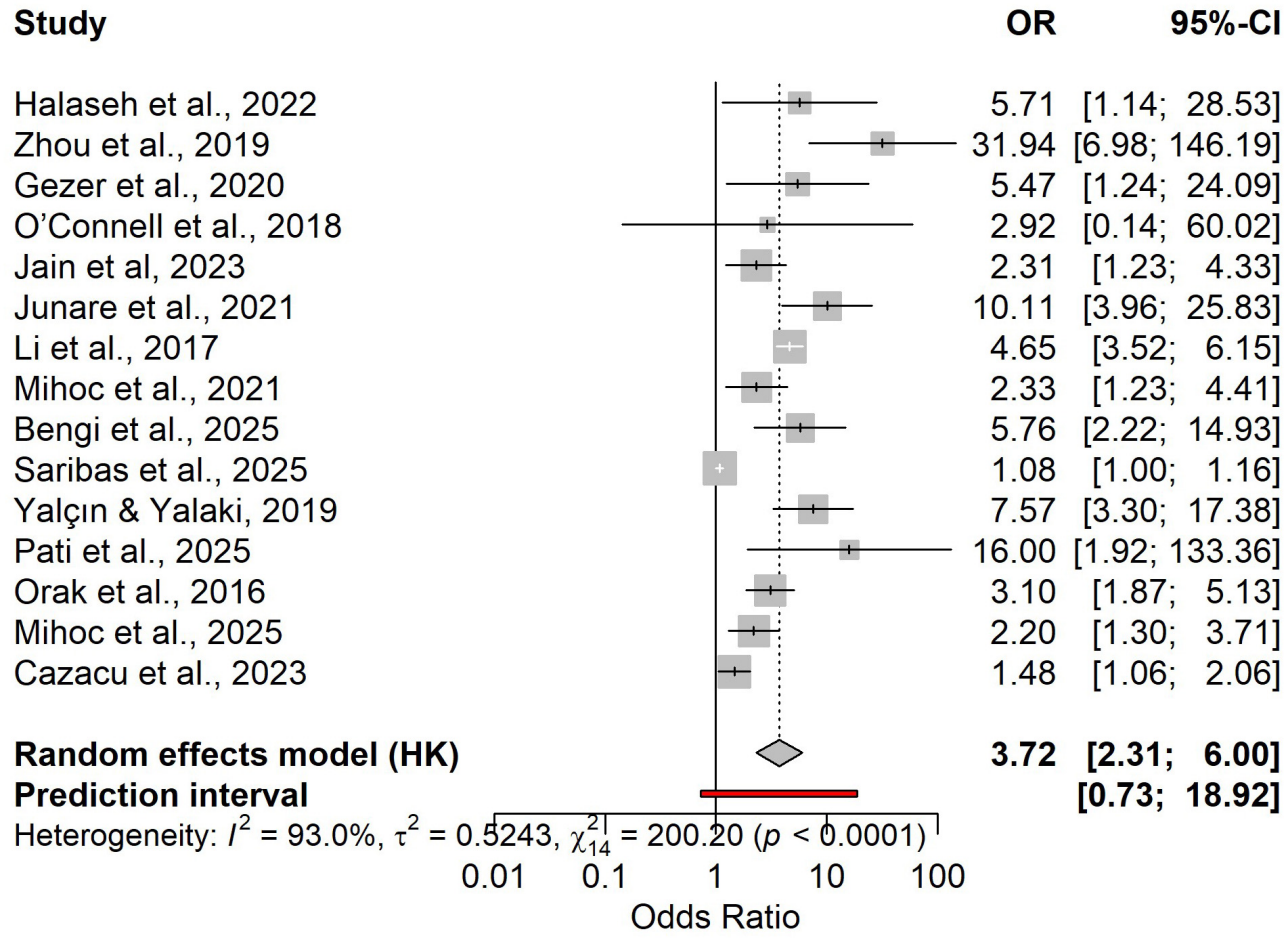
Forest plot of the association between admission NLR and mortality (random-effects model; OR and 95% CI).

**Figure 14:**
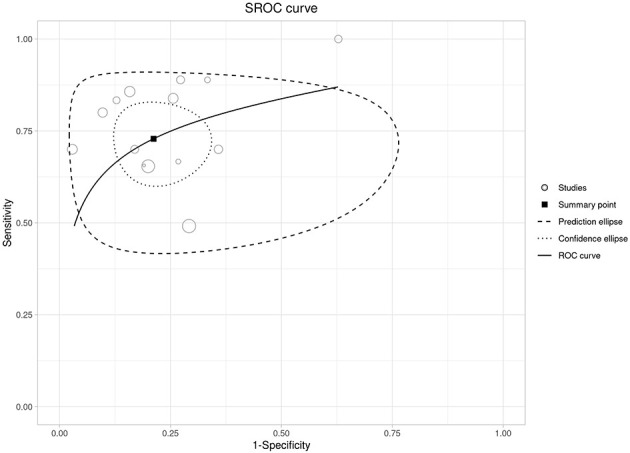
SROC curve for admission NLR in predicting mortality, with the summary point and confidence/prediction ellipse regions.

Across 15 studies, higher admission NLR was associated with increased mortality (pooled OR 3.72), but with very high heterogeneity and a wide prediction interval (Figure 13). In 14 studies with 2 × 2 data (*N* = 4,090), the HSROC analysis showed good overall performance (Se ≈ 0.73; Sp ≈ 0.79; LR+ ≈ 3.44; LR– ≈ 0.34), with moderate-to-high between-study heterogeneity (Figures 14, S40–S42). Discrimination was good (pooled AUC ≈ 0.80; Figures S43, S44). Reported optimal cut-offs varied (~9.7–18.7) but clustered around ~12, which serves as a pragmatic benchmark with local calibration (Figure S42). At low mortality prevalence, PPV remains modest (≈0.18 at 6%; ≈0.28 at 10%), while NPV is high (≈0.98 and ≈0.96, respectively; Table S10 and Figure S45).

## 4 Discussion

In this systematic review and meta-analysis, NLR measured within the first two days of hospitalization provided time-dependent prognostic information in acute pancreatitis. At admission, NLR provided moderate discrimination for severe disease and good discrimination for mortality, whereas performance for infection-related outcomes, driven largely by infected pancreatic necrosis, was only moderate. Importantly, the association-based synthesis for infected pancreatic necrosis (odds ratios) was not robust, reflecting extreme heterogeneity and the small number of available studies. Overall, the evidence supports NLR as a rapid adjunct for early triage and follow-up assessment, rather than a standalone decision rule.

Several included cohorts directly compared NLR with established multivariable scores for predicting severe acute pancreatitis (Table S11), helping to contextualize NLR within routine risk stratification. Across these head-to-head comparisons, admission-based analyses more often favored multivariable scores (e.g., BISAP and APACHE II) for predicting severe disease in multiple cohorts (35, 37, 70). Findings for mortality and organ failure were less consistent: some cohorts reported better discrimination for mortality with NLR than with BISAP (37), whereas others showed the opposite (35). For organ failure, Junare et al. reported slightly better performance for NLR than for BISAP and APACHE II (51). When dynamic testing was available, NLR reassessed at approximately 48 h sometimes matched or exceeded BISAP/APACHE II for both severe disease and mortality (53, 87). However, these comparisons derive from a limited set of heterogeneous studies with variable endpoints and sampling windows and therefore do not justify firm claims of superiority. Taken together, NLR is best interpreted as a universally available complement to established prognostic scores, with its main value in rapid early screening and short-interval reassessment during the first forty-eight hours.

Biologically, NLR captures two early processes in acute pancreatitis: neutrophil-driven innate activation (DAMP/cytokine/NET-mediated injury) and relative lymphopenia reflecting stress-related immune dysregulation. This dual signal supports its use as an early, readily available marker during the first days of illness (93) and aligns with evidence linking neutrophils/NETs to systemic complications in AP (8–10, 94). Overall, NLR condenses the early inflammatory–immune balance into a simple, readily repeatable metric, making 0–48-h trends clinically informative. This is consistent with guideline emphasis on early stratification aimed at predicting persistent organ failure, infected pancreatic necrosis, and mortality (1, 2). Across diverse conditions, higher NLR is associated with worse outcomes, reflecting the broader prognostic relevance of neutrophilia with relative lymphopenia (95). In AP, many cohorts report higher NLR in patients who progress to organ dysfunction, infection, and death; however, thresholds and sampling windows vary, reinforcing the need for dynamic, context-aware interpretation rather than reliance on a single universal cut-off (1, 2).

To address this variability, we performed a prespecified systematic review using two complementary lenses: (i) association measures (OR/RR) and (ii) prognostic accuracy (ROC/HSROC). This approach allowed us to assess both the direction of risk and the practical discriminatory value of NLR across clinically relevant early time windows. Consistent with the evolving inflammatory response, NLR measured at ~24 h generally showed improved discrimination compared with admission. For day-1 reassessment, many cohorts support a pragmatic working threshold near ~8, best used for serial monitoring rather than as a standalone decision rule. By ~48 h, reported thresholds tend to shift lower (often around ~7, commonly within 6–8), supporting continued serial reassessment. Lower or declining values in clinically stable patients may help justify de-escalation, whereas persistently elevated or rising values should prompt evaluation for evolving organ dysfunction or complications.

Similarly, emergency-department cohorts suggest that an admission NLR of approximately 9–10 can aid early identification of patients unlikely to develop a severe course and may outperform single routine laboratory measures (e.g., CRP, creatinine, BUN) in the first hours—supporting a rule-out–oriented role rather than definitive rule-in (28, 96). In multivariable models incorporating routine hemogram parameters, NLR often remains among the strongest contributors to ICU admission and organ failure, supporting its relevance for predicting persistent organ failure rather than only binary severity classification (28, 51).

Etiology may modify performance. In biliary AP, admission NLR tends to show higher sensitivity than specificity, suggesting stronger rule-out than rule-in utility, and limited data in hypertriglyceridemic AP suggest that practical admission thresholds often fall toward the upper end of the ~9–11 range. Across etiologies, confounding by comorbidities, medications, resuscitation intensity, and antibiotic strategies remains important, yet few studies have formally quantified clinical utility (e.g., via decision-curve analysis). Importantly, etiology-specific evidence, particularly for alcohol-induced acute pancreatitis, remains scarce. None of the included studies were specifically designed to evaluate NLR in alcoholic AP, nor did they report sufficiently detailed, etiology-stratified diagnostic/performance metrics to enable a separate quantitative synthesis. At present, only a single dedicated report has addressed NLR/PLR in alcoholic AP (97), which precludes meta-analysis and underscores the need for well-designed prospective studies with standardized etiology-stratified reporting.

For fatal outcomes, pooling the available ROC estimates in our meta-analysis yielded a summary AUC = 0.80 at admission and a weighted mean = 0.79–0.80, which we deem sufficient to use the NLR as an early risk “signal,” with subsequent confirmation by more complex scores and specialized biomarkers. For IPN, the OR-based association meta-analysis was non-robust (imprecise pooled estimate with extreme heterogeneity across only four studies). Therefore, NLR should not be interpreted as a consistent prognostic factor for IPN; its role in infection-related endpoints is best viewed as adjunctive and interpreted within diagnostic accuracy metrics and dynamic clinical assessment. Accordingly, it should complement (not replace) PCT/CRP and imaging.

We emphasize that for early detection of a severe course, cytokine markers, particularly IL-6, demonstrate the highest diagnostic informativeness among single tests (Se = 87% and Sp = 88% at a threshold >50 pg/ml in the first hours), whereas procalcitonin performs best for infected necrosis (dynamic high LR+), and CRP is more useful as a background indicator of systemic inflammatory activity (13, 98). Therefore, after 72 h and thereafter, and particularly in clinically unstable patients, it is rational to rely on PCT-based algorithms, using the NLR as a low-cost indicator of trend. Where available, early IL-6 may outperform NLR for confirming severe disease; however, it is less accessible in routine practice. In this context, NLR can serve as an immediate first-line screen, complemented by PCT/CRP, composite scores, and imaging, consistent with professional society recommendations (1, 2).

Clinical translation of NLR is best framed as serial assessment (Box 1). At admission, a higher NLR can flag patients who warrant closer monitoring, whereas a low NLR, alongside clinical stability, argues against immediate escalation. Repeat testing at ~24–48 h adds context: falling values support de-escalation, while persistently high or rising values should prompt reassessment for organ dysfunction and complications and consideration of additional biomarkers when available.

Box 1 **NLR clinical interpretation (0–48 h)**

NLR is an adjunct screening signal available immediately from the complete blood count.

Best suited for early triage and serial reassessment (day 0 → day 1 → day 2), not for a single stand-alone decision.

Because thresholds vary across settings, local calibration and integration with clinical scores/labs are required.

NLR tends to be more useful for ruling out an early high-risk course when low (high NPV in many settings), while rule-in performance is only moderate.

Comorbidities, medications, and intercurrent infection can confound NLR; interpret in context.

## 5 Limitations

The present findings should be interpreted in light of several methodological and clinical constraints. Substantial between-study heterogeneity persists; differences in study design, cohort composition, etiologic spectrum of acute pancreatitis, timing of blood sampling, and analytical platform characteristics inevitably influence the accuracy and reproducibility of the metric. Variability in threshold values reflects not only the statistical methods used to derive them, but also the clinical context of application—event prevalence, initial treatment strategies, criteria for severity and complications, and divergent operational definitions of endpoints. Additional risks of bias include incomplete primary data, selective reporting, lack of prespecified analytical protocols, and the limited number of prospective studies using standardized measurement procedures. Residual confounding inherent to observational research remains a major challenge, encompassing comorbidities, concomitant pharmacotherapy, the intensity of fluid resuscitation, and antibacterial prophylaxis. Finally, most studies do not provide access to individual patient data and rarely evaluate clinical utility (e.g., decision-curve analysis), which constrains interpretability, external validity, and translation of results into real-world care pathways.

## 6 Future perspectives

Prospective multicenter validation should be prioritized, with uniform endpoint definitions, harmonized protocols for sample collection and processing, and pre-specified clinical thresholds that allow local recalibration. Systematic evaluation of early marker dynamics—within the first hours and days—as triggers for intensified monitoring and treatment is a promising direction, as is integration into multimodal prognostic models that combine clinical features, routine biomarkers, and imaging with modern analytical tools. Traditional diagnostic metrics should be complemented by assessments of net clinical benefit, effects on decision-making, resource requirements, and cost-effectiveness across diverse healthcare systems. Priorities also include open reporting, replication in independent cohorts, adoption of transparent standards for the development and validation of prognostic tools, and implementation studies that test readiness for routine practice while ensuring equitable access to innovative approaches across diverse clinical settings.

## 7 Conclusions

This systematic review and meta-analysis suggest that the neutrophil-to-lymphocyte ratio measured within the first 48 h of hospitalization may provide clinically useful, low-cost prognostic information in acute pancreatitis when used as an adjunct to established scores and routine assessment (Box 1). Across the included studies, NLR showed moderate-to-good discrimination for severe disease at presentation (pooled AUC ~0.77), with commonly reported thresholds shifting over time (approximately ~9 at admission, ~8 at 24 h, and ~7 at 48 h), reflecting the evolving inflammatory response. However, substantial between-study heterogeneity, variable endpoint definitions, and differences in patient mix and sampling windows limit the generalizability of any single cut-off. Therefore, NLR should not be used as a stand-alone criterion; rather, it may support early screening and serial reassessment within 0–48 h, with mandatory local calibration and integration with validated clinical scores, other laboratory markers, and imaging, where appropriate. Prospective multicenter studies with standardized time windows and prespecified thresholds are needed to determine net clinical benefit and to guide implementation pathways.

## Data Availability

The original contributions presented in the study are included in the article/Supplementary material, further inquiries can be directed to the corresponding author.
