# Supplementary material for: Neutrophil-to-lymphocyte ratio for primary risk stratification in acute pancreatitis: a systematic review and meta-analysis
Source: Front Med (Lausanne). 2026 Jan 13;12:1729339. doi: 10.3389/fmed.2025.1729339 (PMC12835348; doi:10.3389/fmed.2025.1729339)
Supplement: Supplementary file 1 [file Data_Sheet_1.pdf]

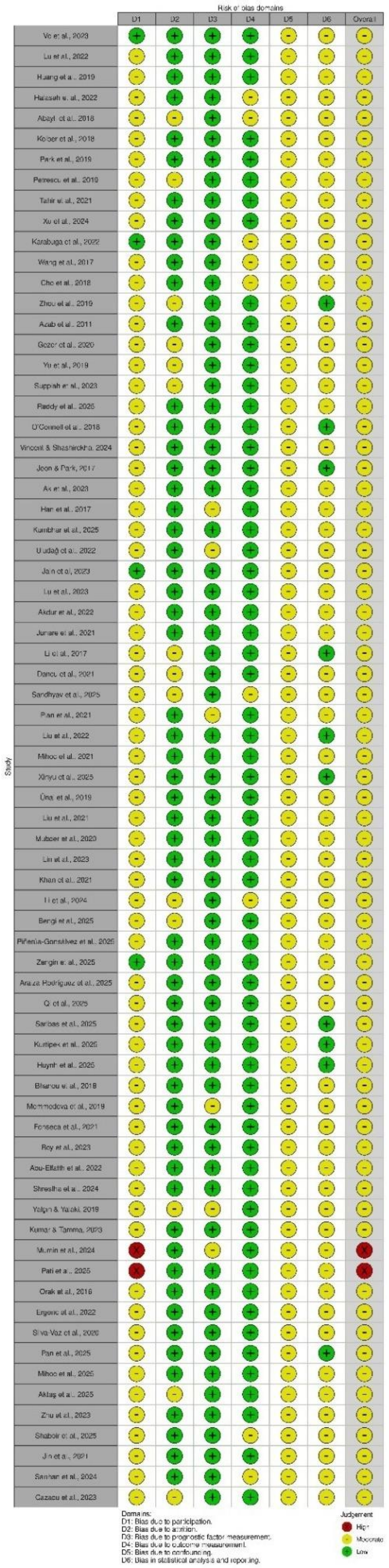

Supplementary Figure 1. Risk of bias by QUIPS across the included studies.

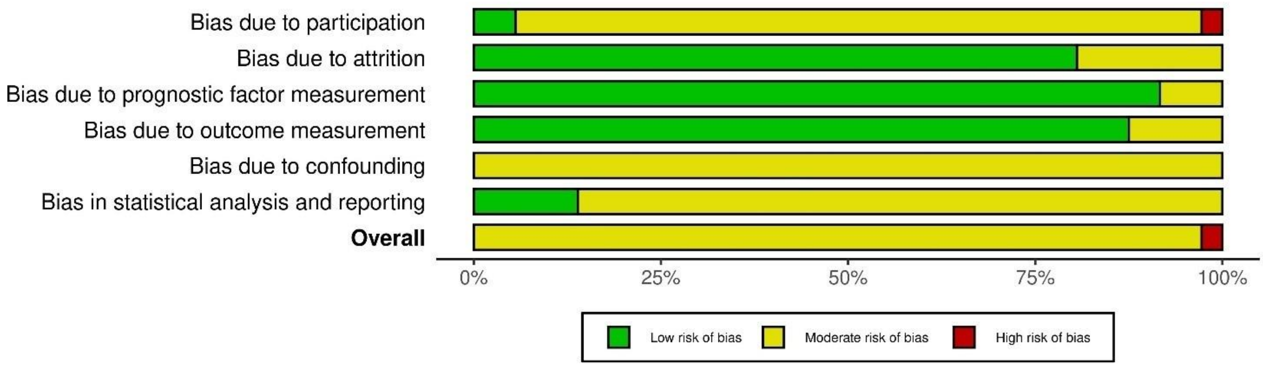

Supplementary Figure 2. Summary of risk of bias by QUIPS domains.

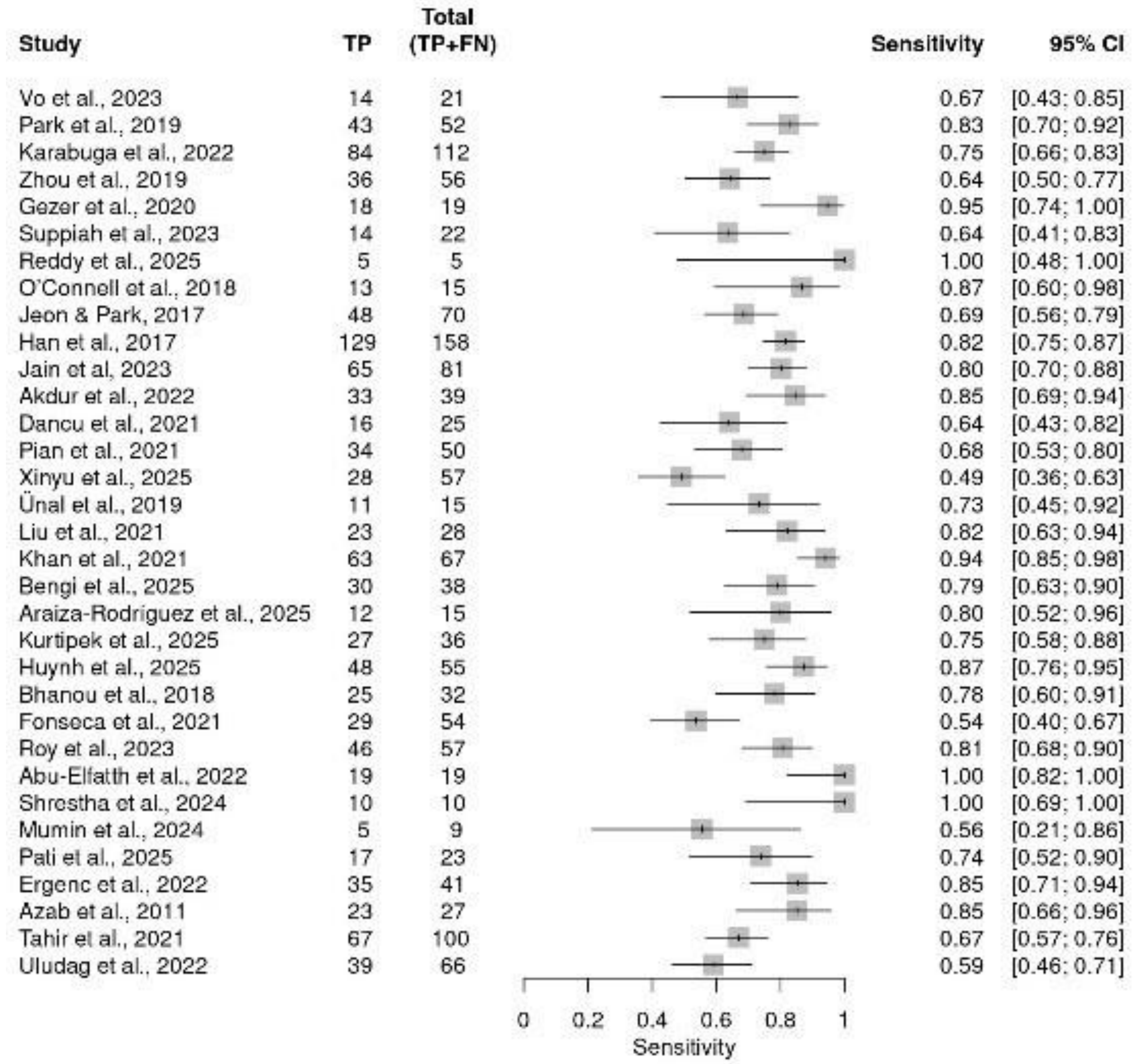

Supplementary Figure 3. Forest plot of individual study sensitivity (NLR on day 0).

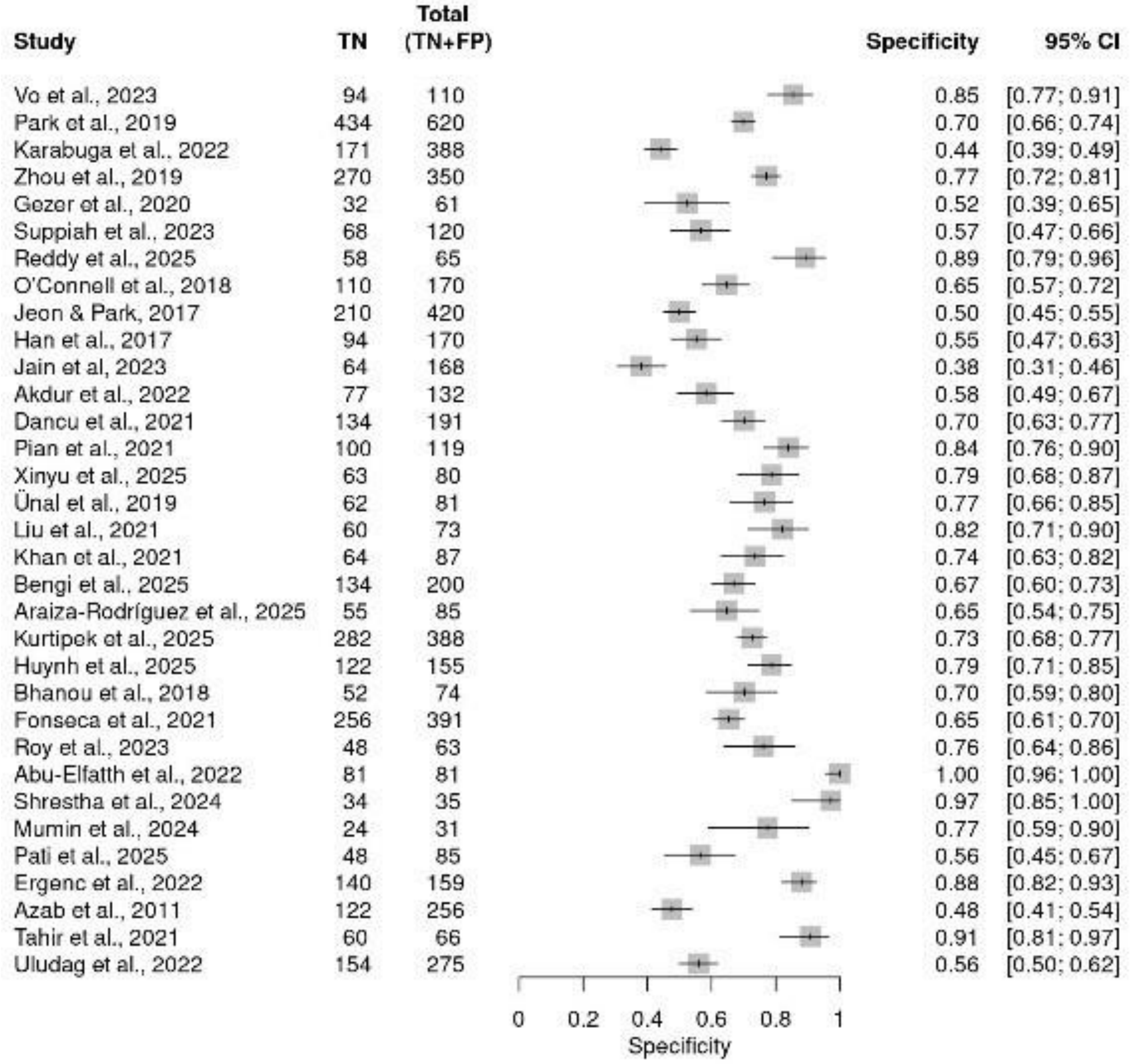

Supplementary Figure 4. Forest plot of individual study specificity (NLR on day 0).

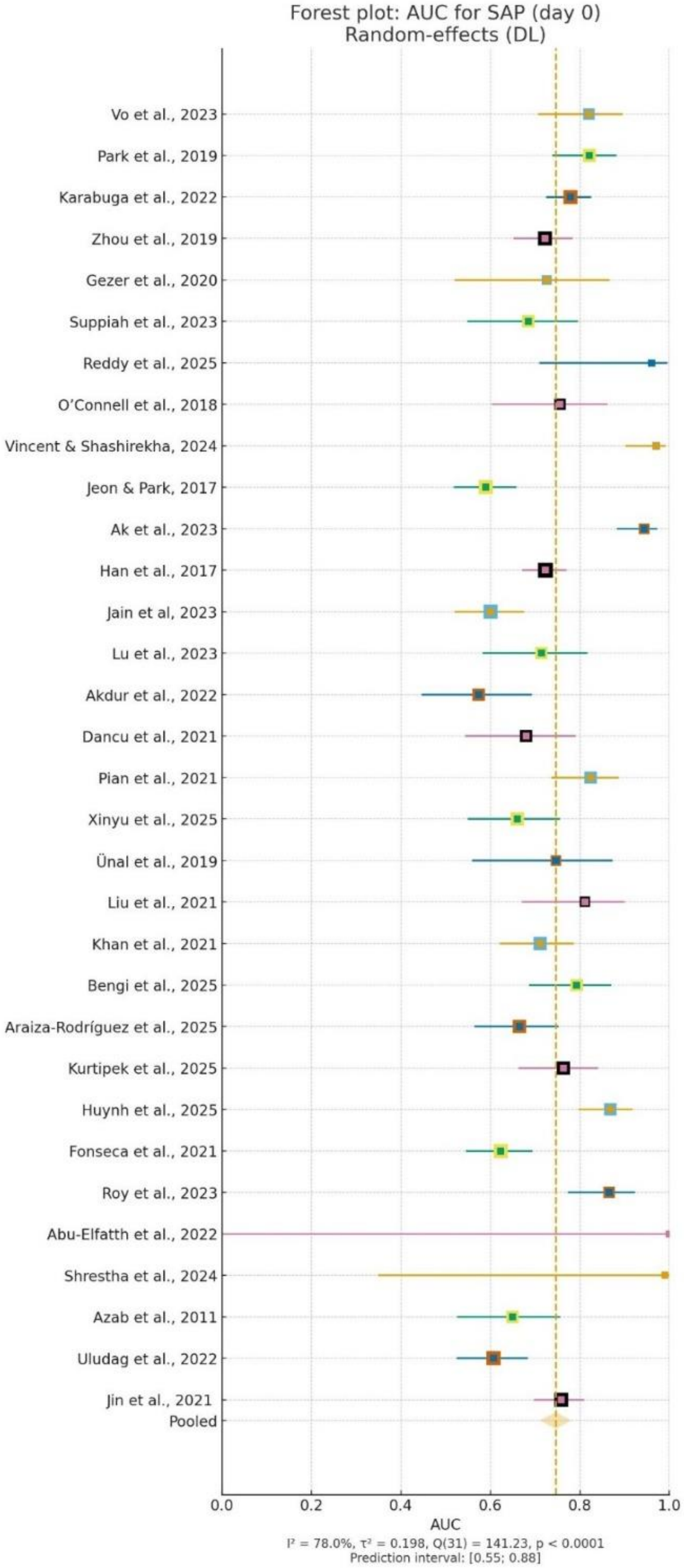

Supplementary Figure 5. Forest plot of AUC for NLR at admission (day 0) with respect to severe acute pancreatitis. Random-effects model; individual AUCs with 95% CIs, the pooled estimate (RE), and heterogeneity statistics (I^2, τ^2) are shown.

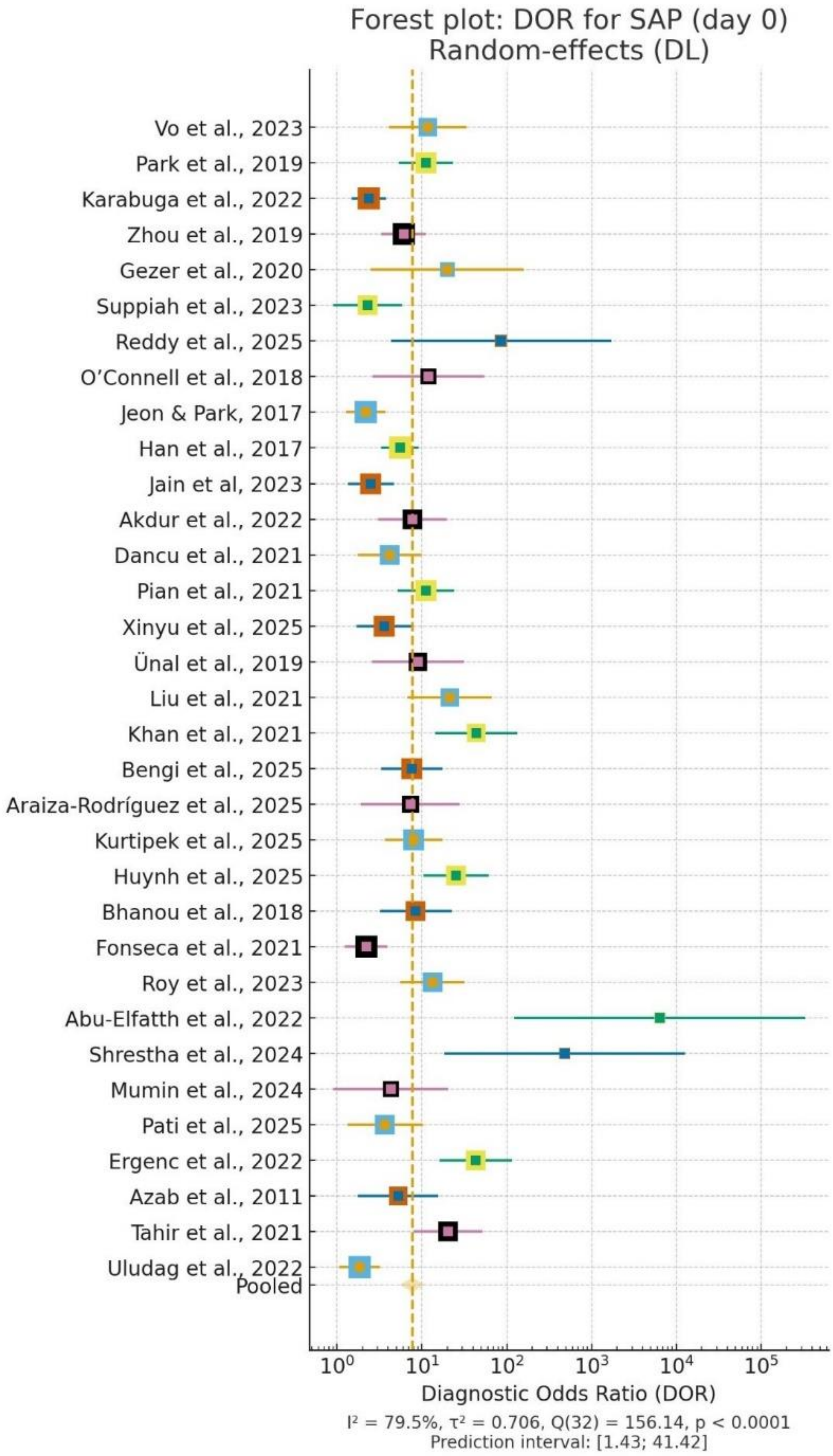

Supplementary Figure 6. Forest plot of log(DOR) for NLR at admission (day 0) with respect to severe acute pancreatitis. Random-effects model; individual estimates with 95% CIs, the pooled estimate (RE), and the 95% prediction interval are shown.

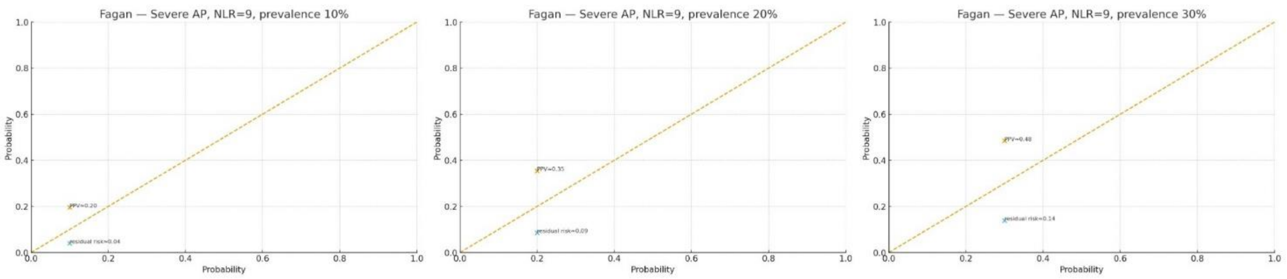

Supplementary Figure 7. Fagan plots – Severe acute pancreatitis, NLR = 9: effect of baseline prevalence (10%, 20%, 30%).

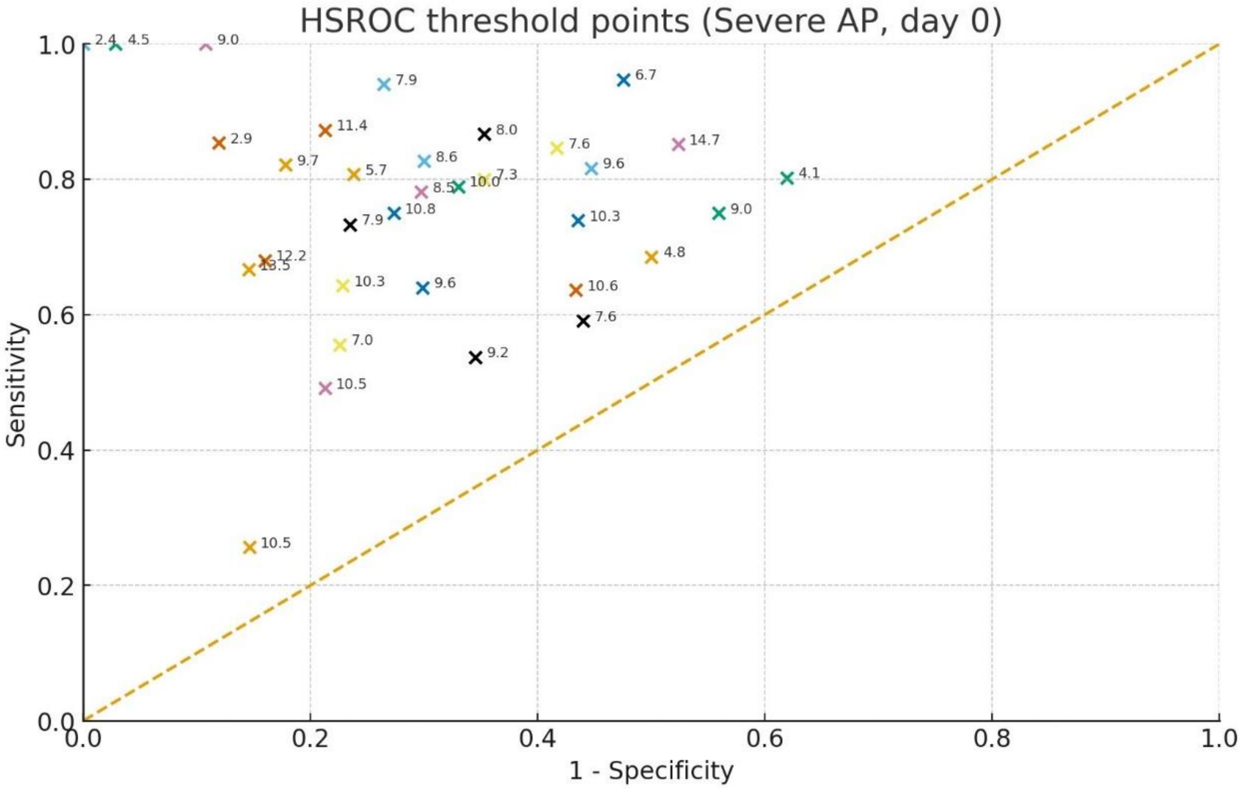

Supplementary Figure 8. HSROC threshold points for NLR at admission (day 0) in severe acute pancreatitis.

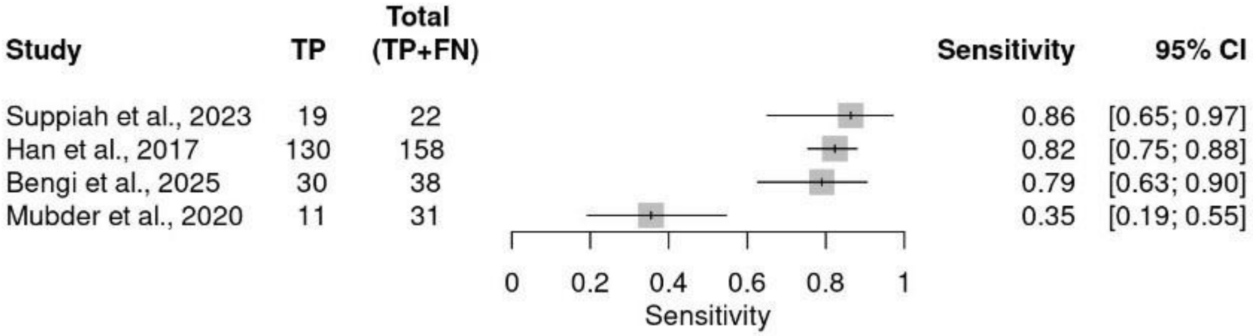

Supplementary Figure 9. Forest plot of individual study sensitivity (NLR on day 1).

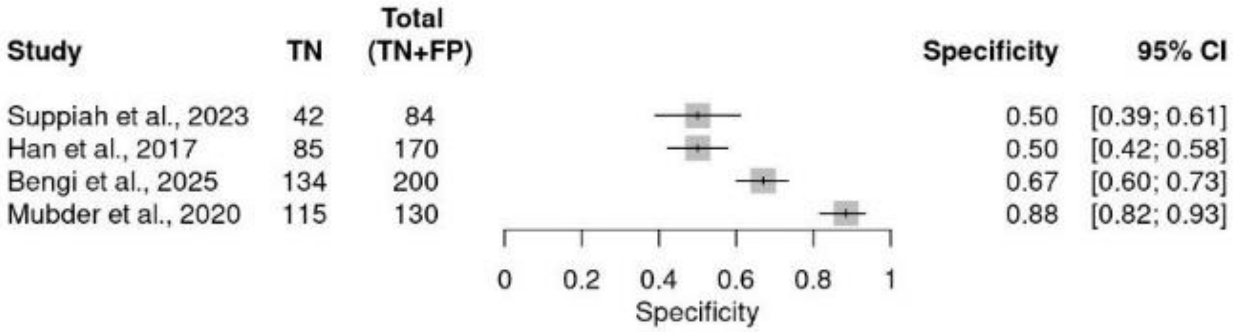

Supplementary Figure 20. Forest plot of individual study specificity (NLR on day 1).

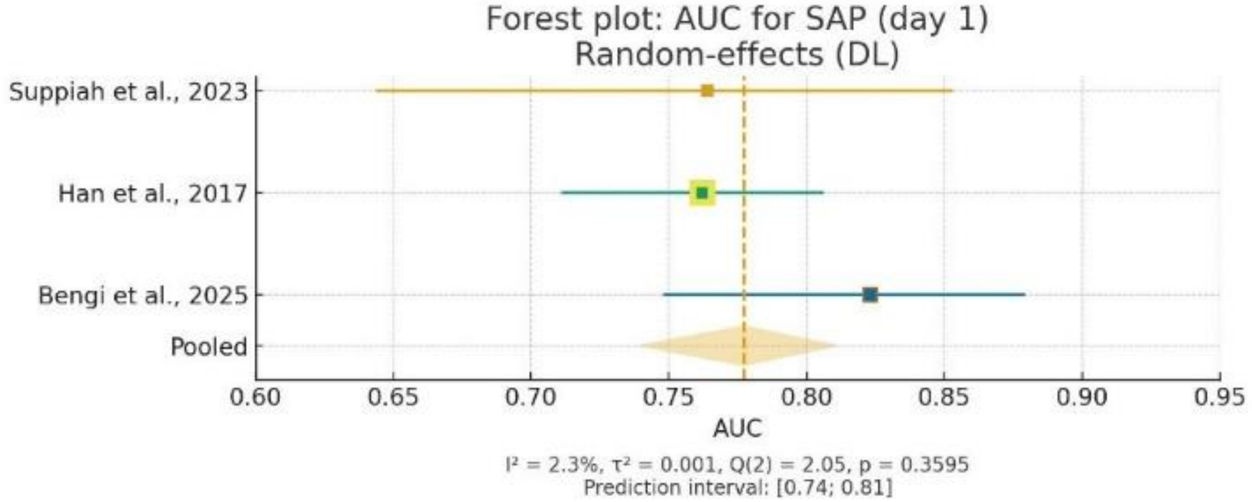

Supplementary Figure 31. Forest plot of AUC for the NLR on day 1 for severe acute pancreatitis. Random-effects model; individual AUCs with 95% CIs, the pooled estimate (RE), and heterogeneity metrics (I^2, τ^2) are shown.

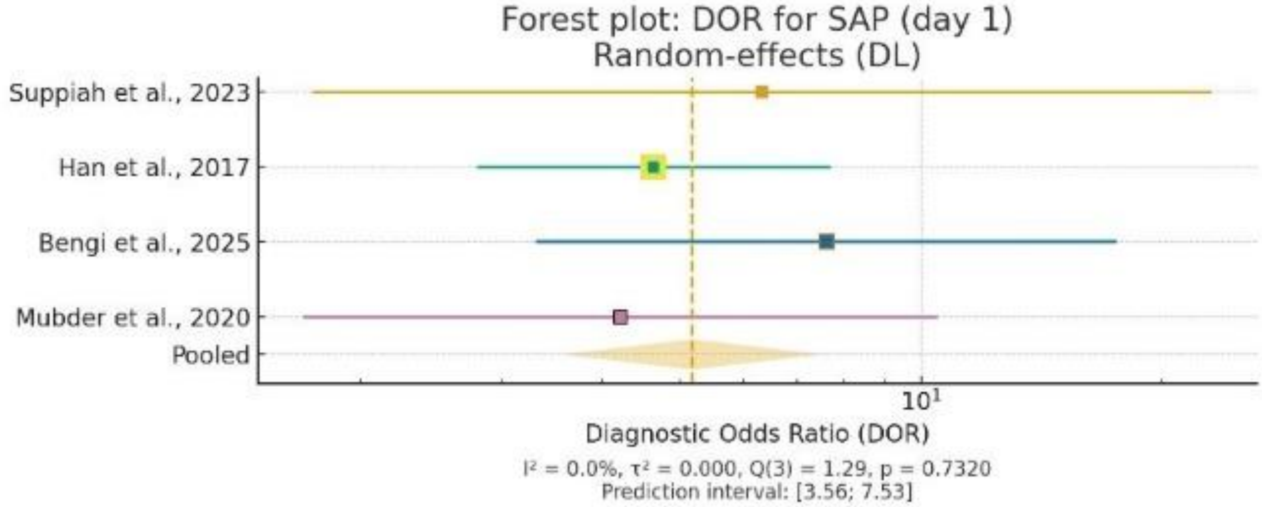

Supplementary Figure 42. Forest plot of log(OR) for the NLR on day 1 for severe acute pancreatitis. Random-effects model; individual estimates with 95% CIs and the pooled estimate (RE) are shown.

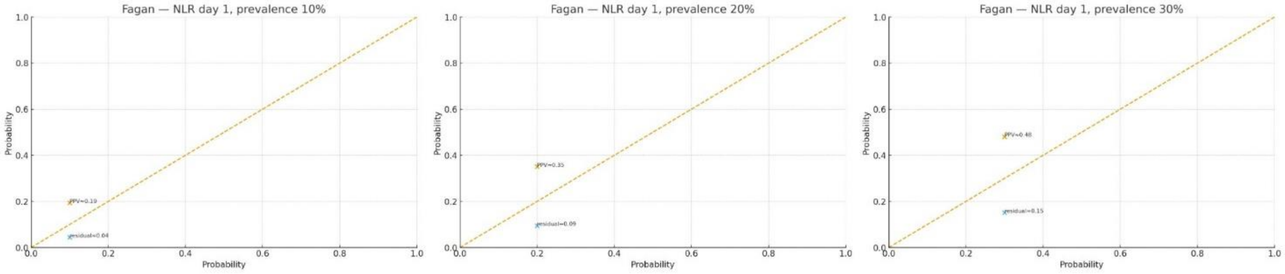

Supplementary Figure 53. Fagan nomograms for day-1 NLR at disease prevalences of 10%, 20%, and 30% for predicting severe acute pancreatitis.

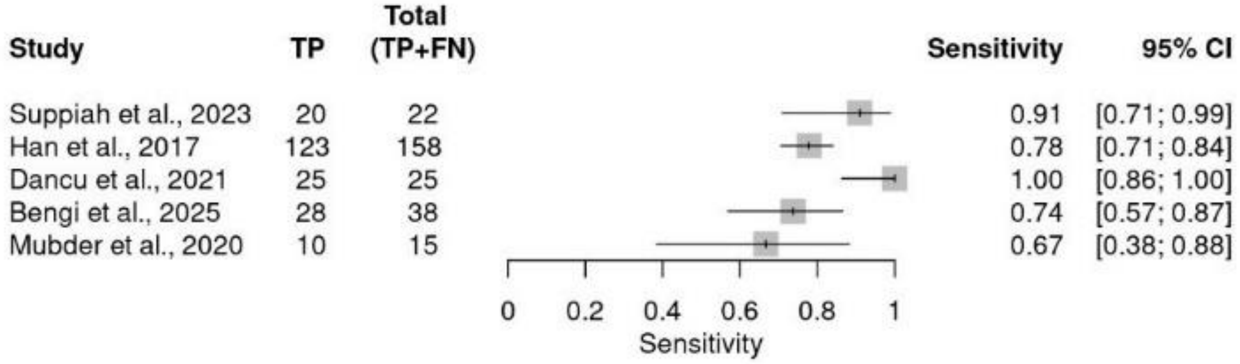

Supplementary Figure 64. Forest plot of individual study sensitivity (NLR on day 2).

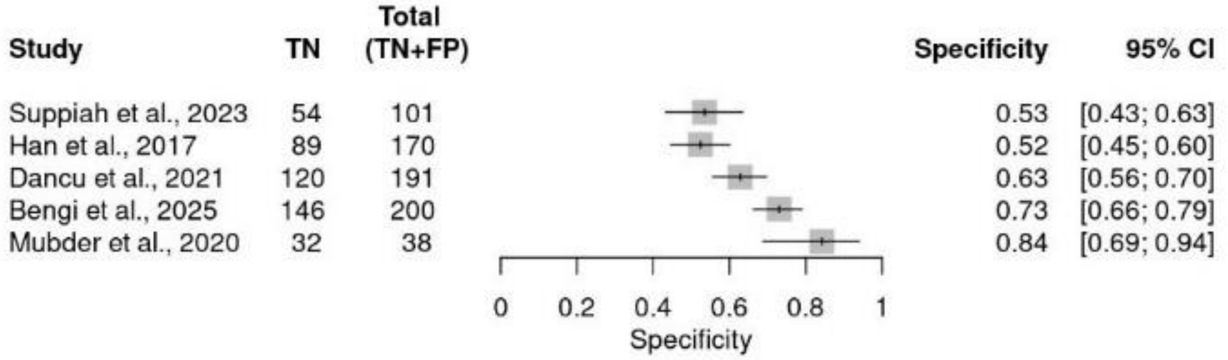

Supplementary Figure 75. Forest plot of individual study specificity (NLR on day 2).

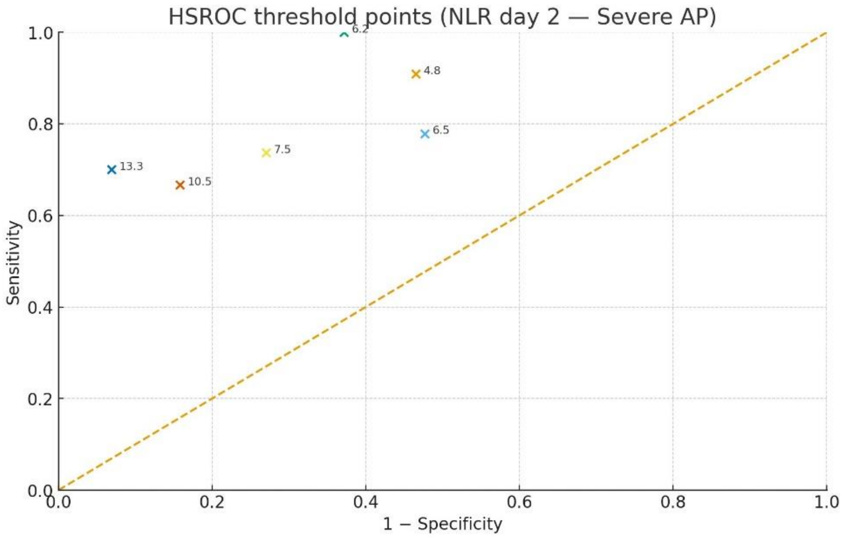

Supplementary Figure 86. HSROC threshold points for day-2 NLR in predicting severe acute pancreatitis.

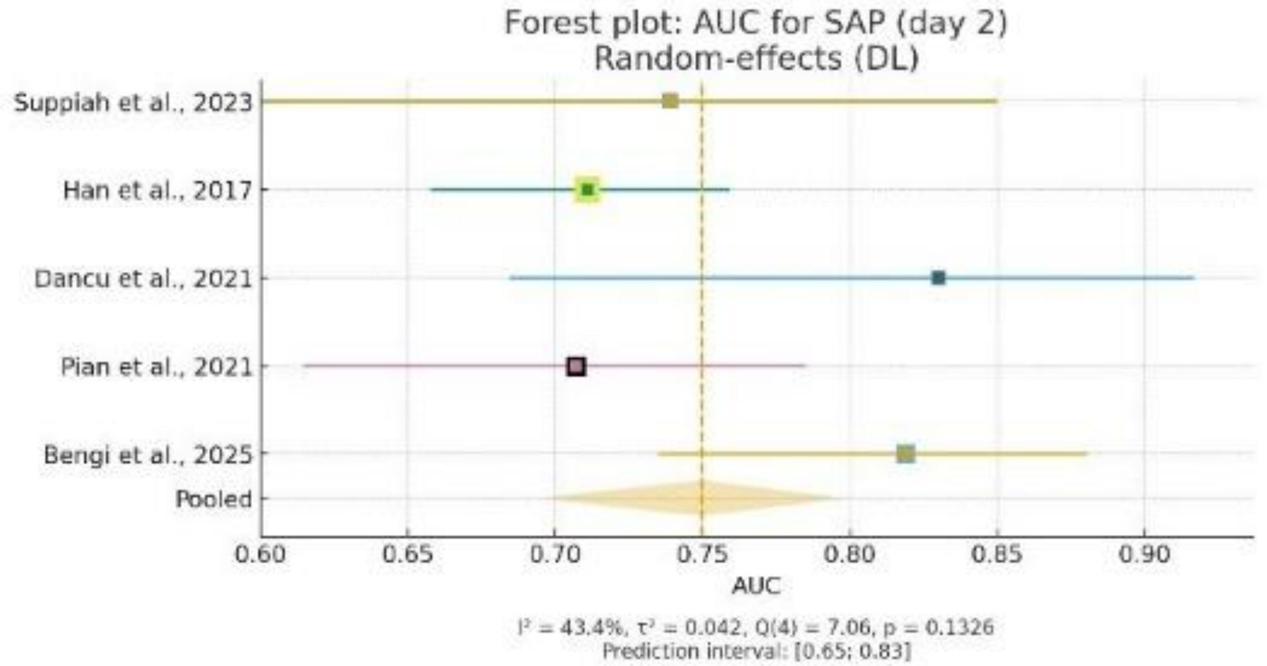

Supplementary Figure 97. Forest plot of AUC for the NLR on day 2 for severe acute pancreatitis. Random-effects model; individual AUCs with 95% CIs, the pooled estimate (RE), and heterogeneity metrics (I^2, τ^2) are shown.

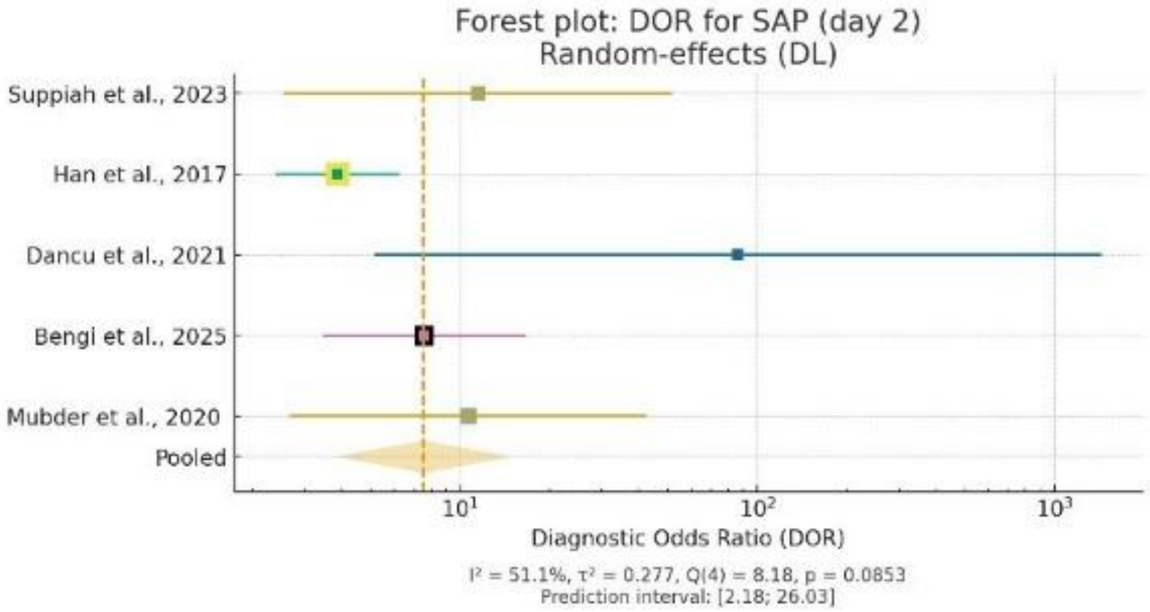

Supplementary Figure 108. Forest plot of log(OR) for the NLR on day 2 for severe acute pancreatitis. Random-effects model; individual estimates with 95% CIs and the pooled estimate (RE) are shown.

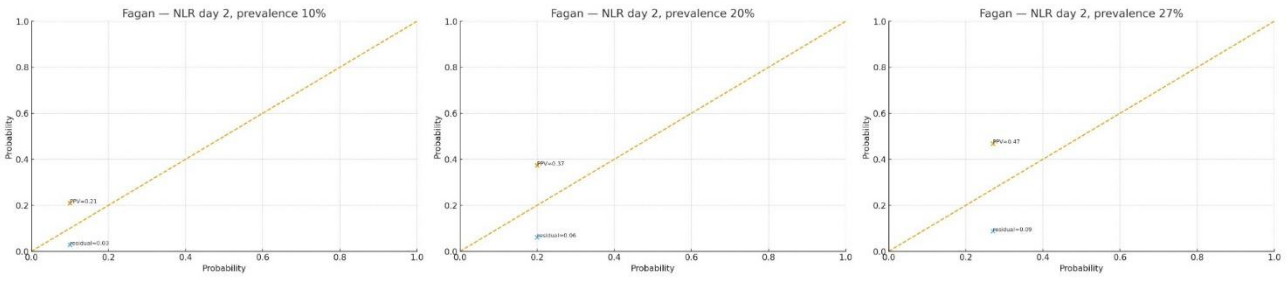

Supplementary Figure 119. Fagan nomograms for day-2 NLR at disease prevalences of 10%, 20%, and 27% for predicting severe acute pancreatitis.

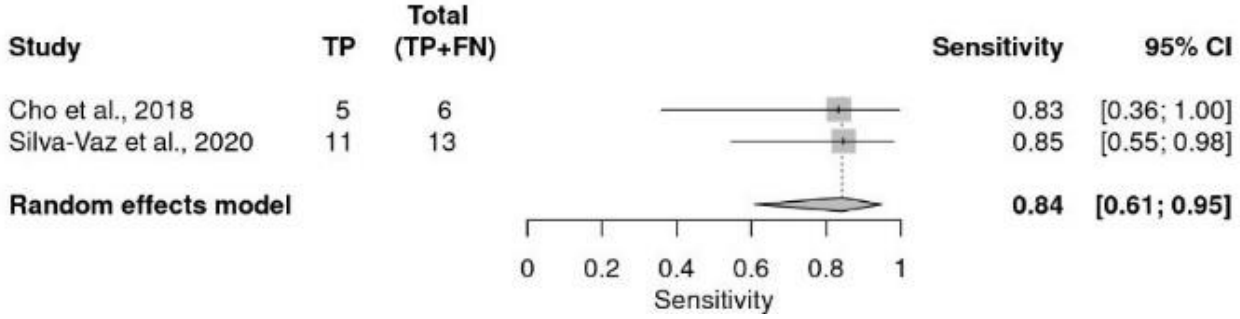

Supplementary Figure 20. Summary forest plot of the sensitivity of NLR at admission for predicting severe biliary pancreatitis.

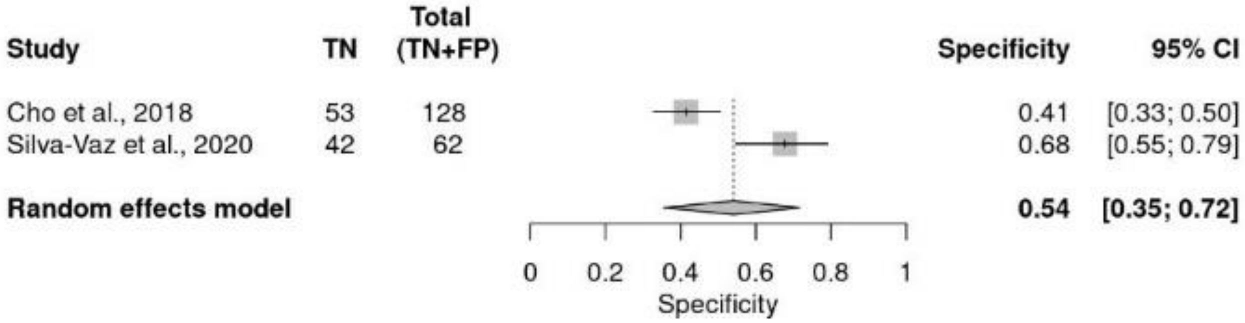

Supplementary Figure 21. Summary forest plot of the specificity of NLR at admission for predicting severe biliary pancreatitis.

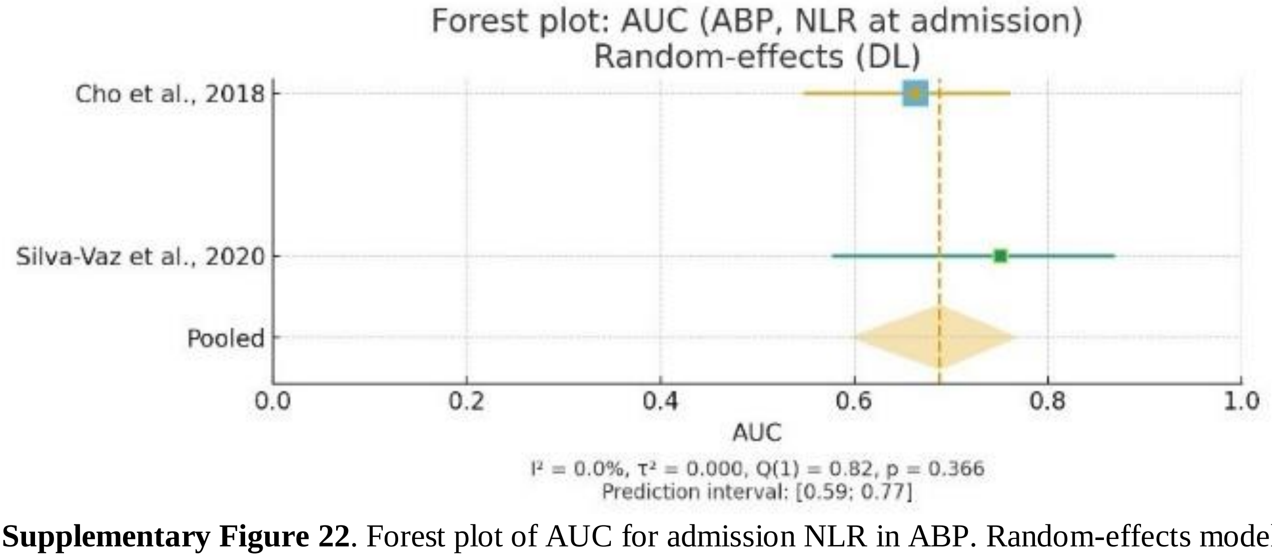

Supplementary Figure 22. Forest plot of AUC for admission NLR in ABP. Random-effects model; individual AUC values with 95% CIs and the pooled estimate (RE) are shown, with heterogeneity statistics (I^2, τ^2) reported.

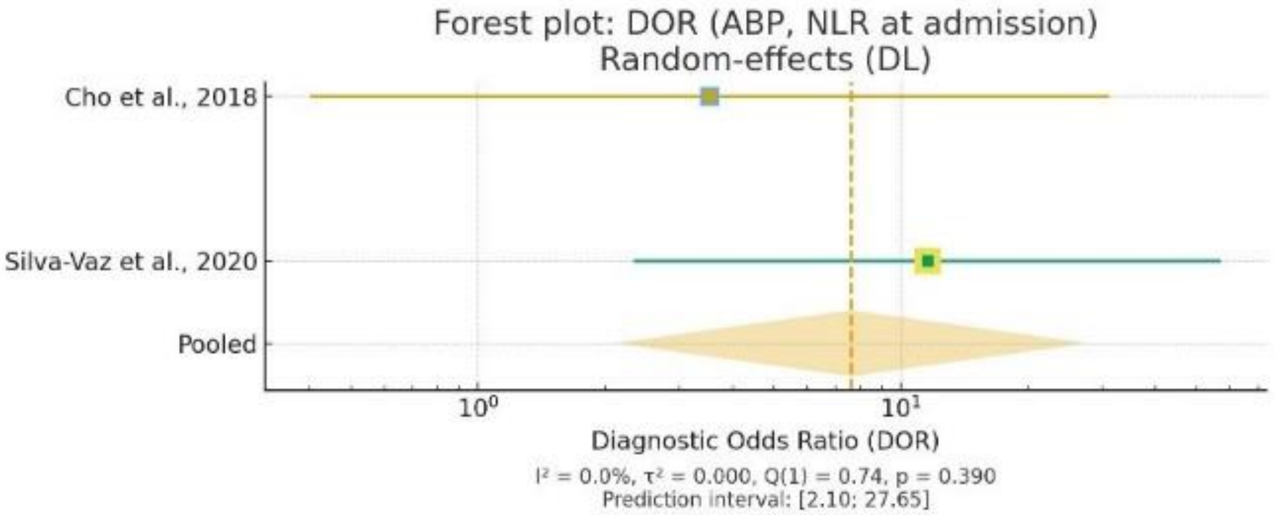

Supplementary Figure 23. Forest plot of log(DOR) for NLR at admission (acute biliary pancreatitis, ABP). Random-effects model; individual study estimates with 95% CIs, the pooled estimate (RE), and the 95% prediction interval are shown.

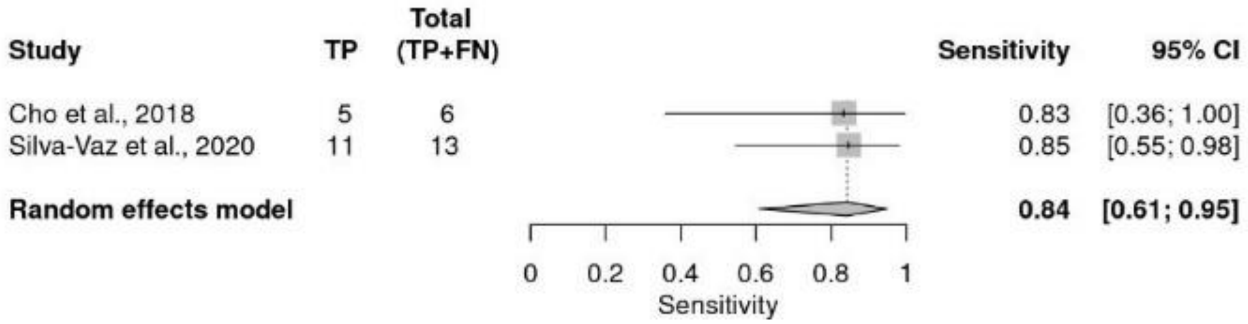

Supplementary Figure 24. Summary forest plot of the sensitivity of NLR at admission for predicting severe biliary pancreatitis.

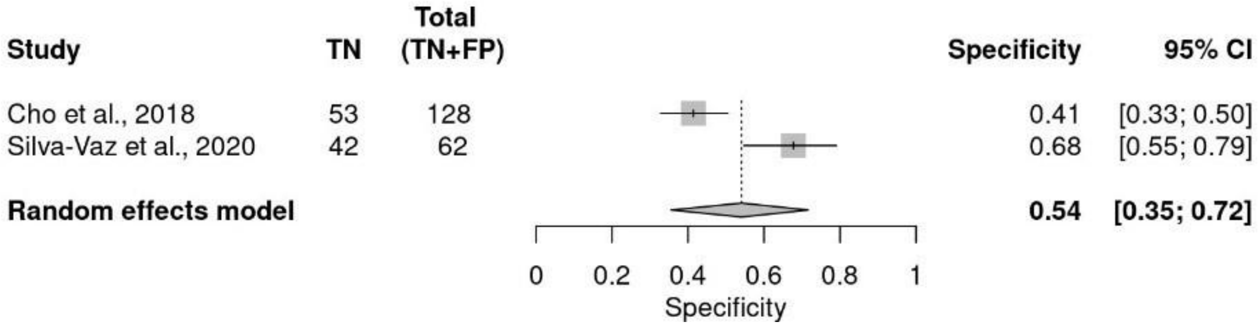

Supplementary Figure 25. Summary forest plot of the specificity of NLR at admission for predicting severe biliary pancreatitis.

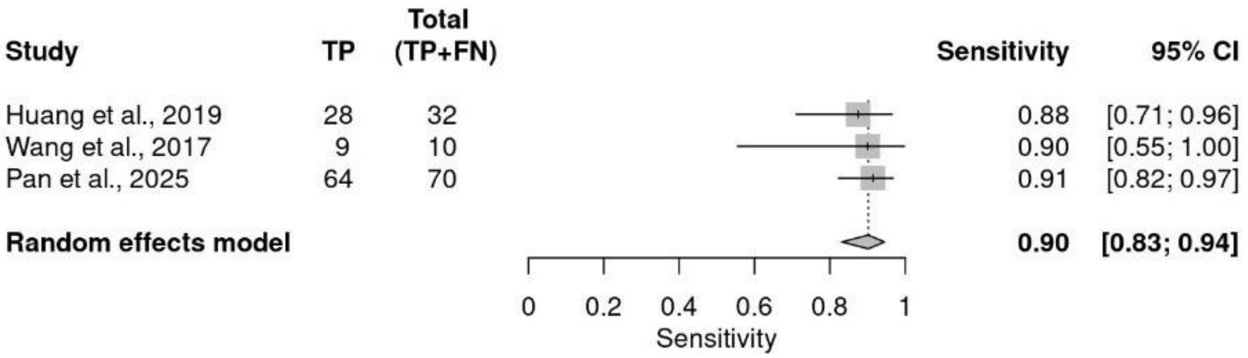

Supplementary Figure 26. Summary forest plot of the sensitivity of NLR at admission for predicting a severe course of hypertriglyceridemia-induced acute pancreatitis.

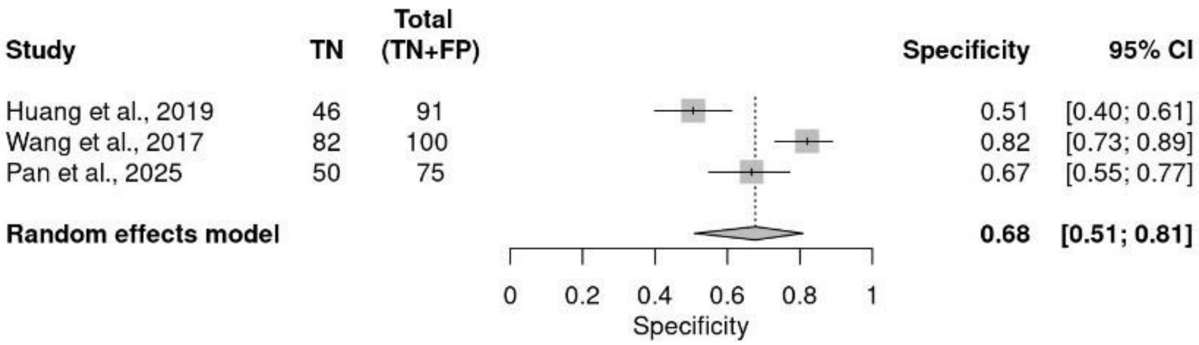

Supplementary Figure 27. Summary forest plot of the specificity of NLR at admission for predicting a severe course of hypertriglyceridemia-induced acute pancreatitis.

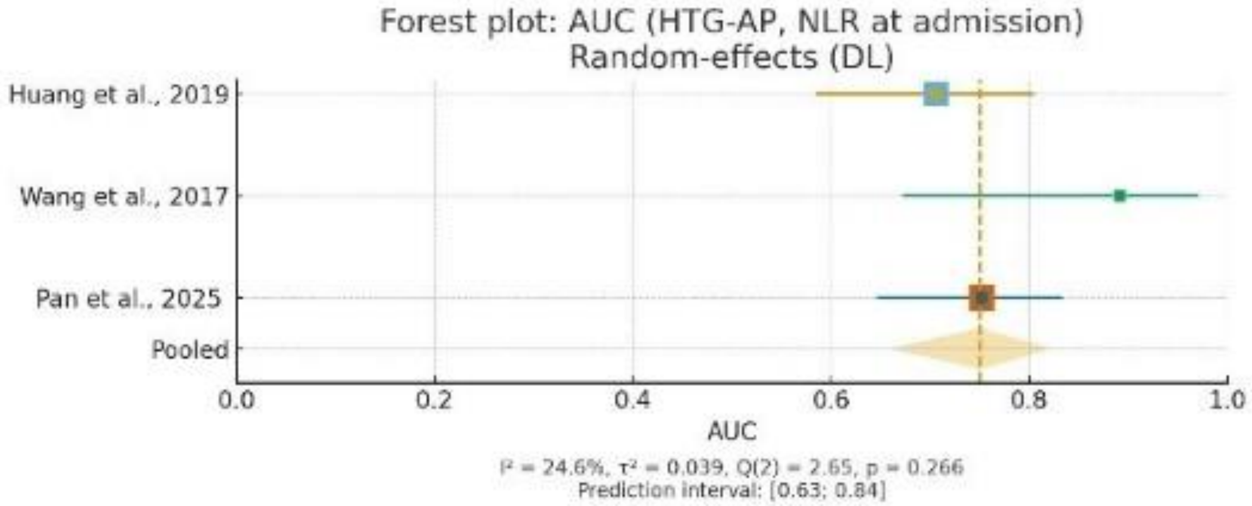

Supplementary Figure 28. Forest plot of AUC for admission NLR in HTG-AP. Random-effects model; individual AUC estimates with 95% CIs and the pooled estimate (RE) are shown, with heterogeneity statistics (I^2, τ^2) reported.

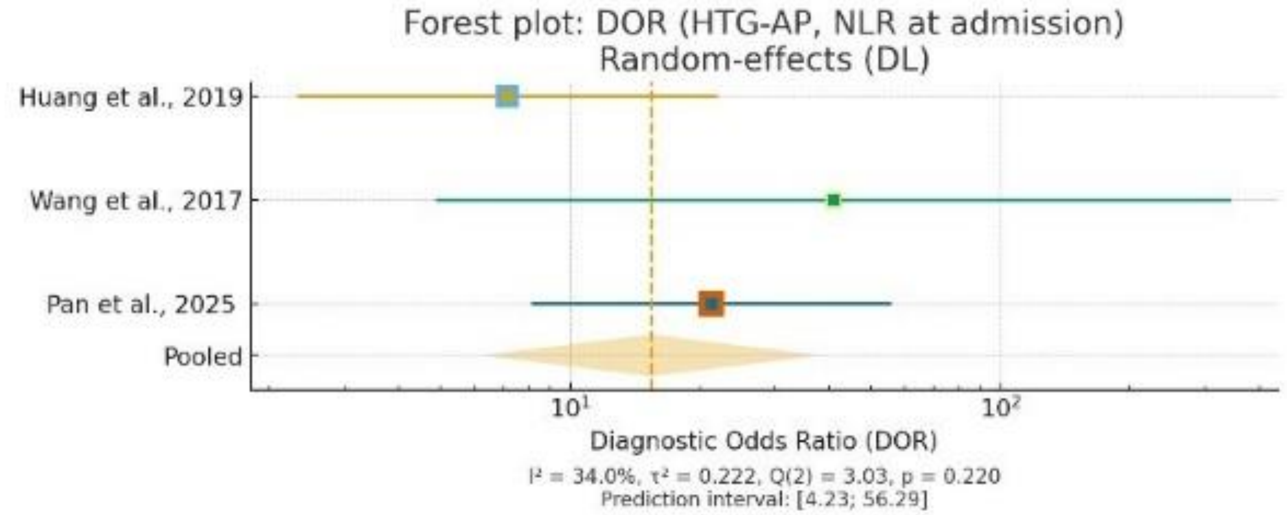

Supplementary Figure 29. Forest plot of log(DOR) for NLR at admission (HTG-AP). Random-effects model; individual study estimates with 95% CIs, the pooled estimate (RE), and the 95% prediction interval are shown.

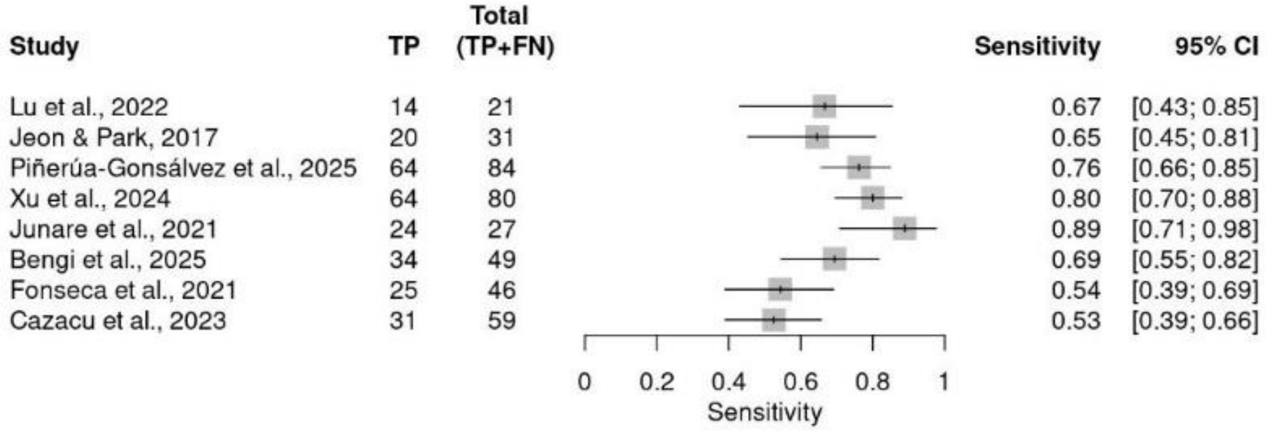

Supplementary Figure 30. Forest plot of the sensitivity of NLR at admission for predicting POF across the included studies.

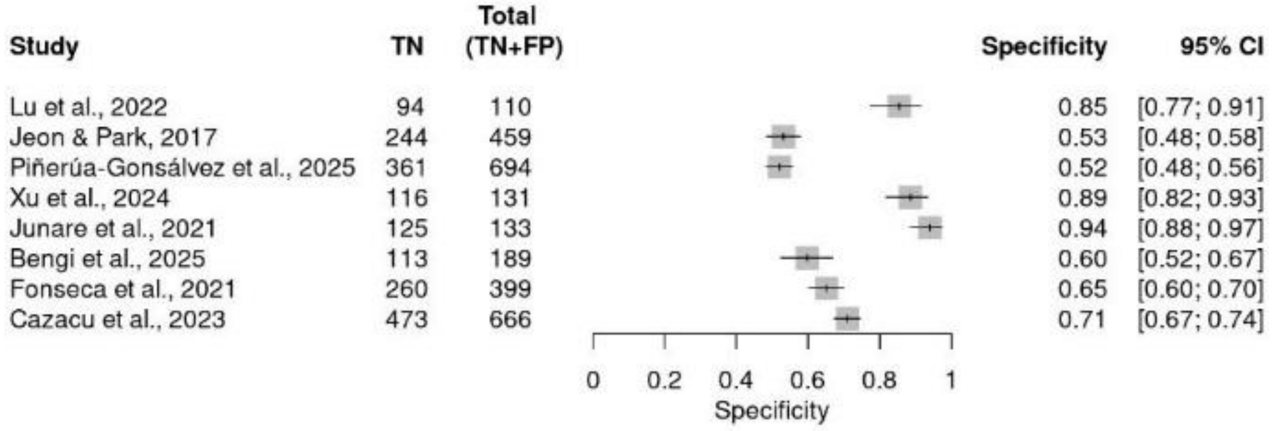

Supplementary Figure 31. Forest plot of the specificity of NLR at admission for predicting POF across the included studies.

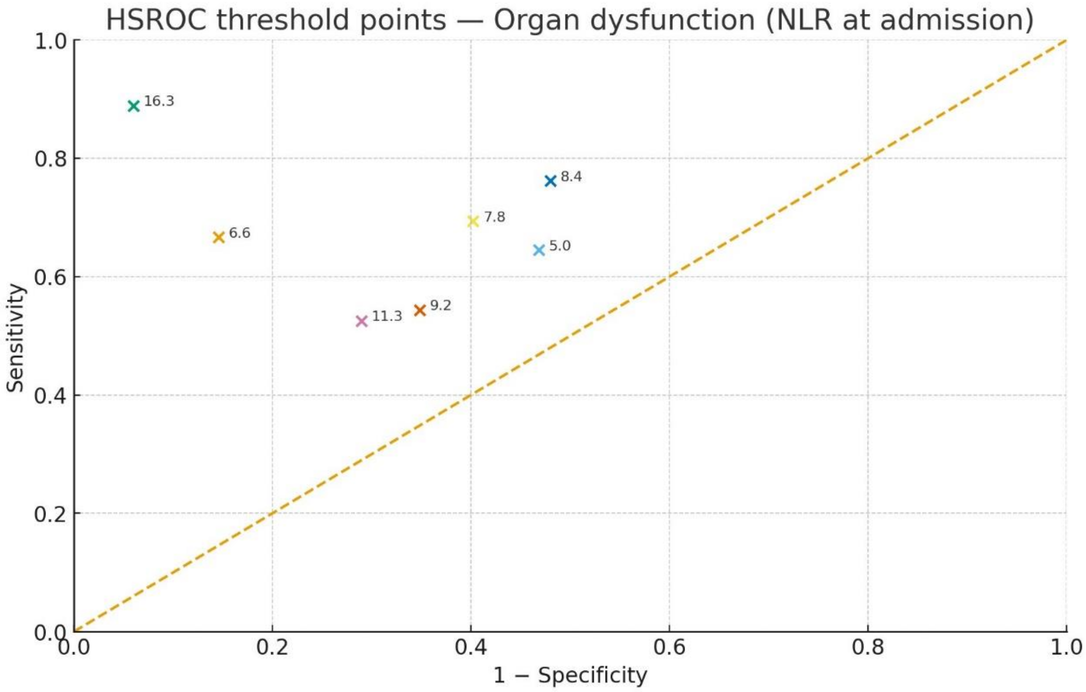

Supplementary Figure 32. HSROC threshold points for organ dysfunction (NLR at admission).

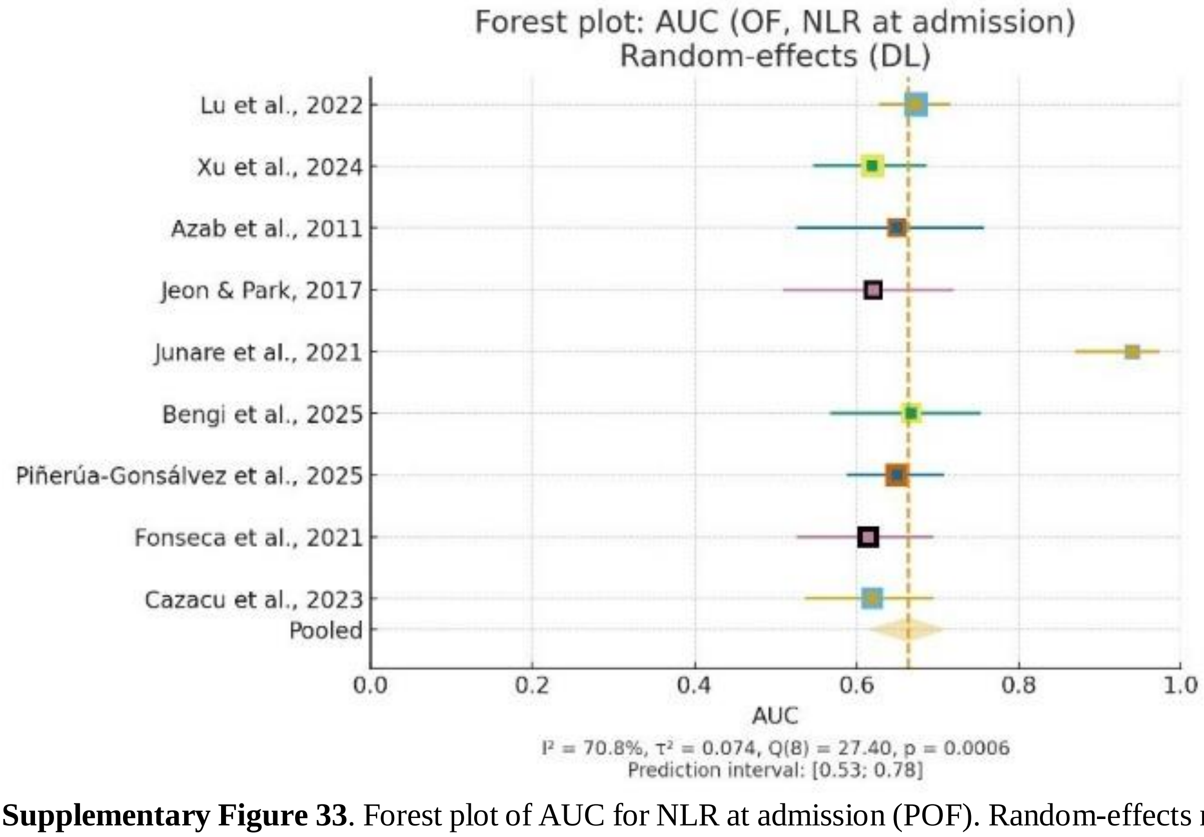

Supplementary Figure 33. Forest plot of AUC for NLR at admission (POF). Random-effects model; individual AUC values with 95% CIs and the pooled estimate (RE) with heterogeneity indicators (I^2, τ^2) are shown.

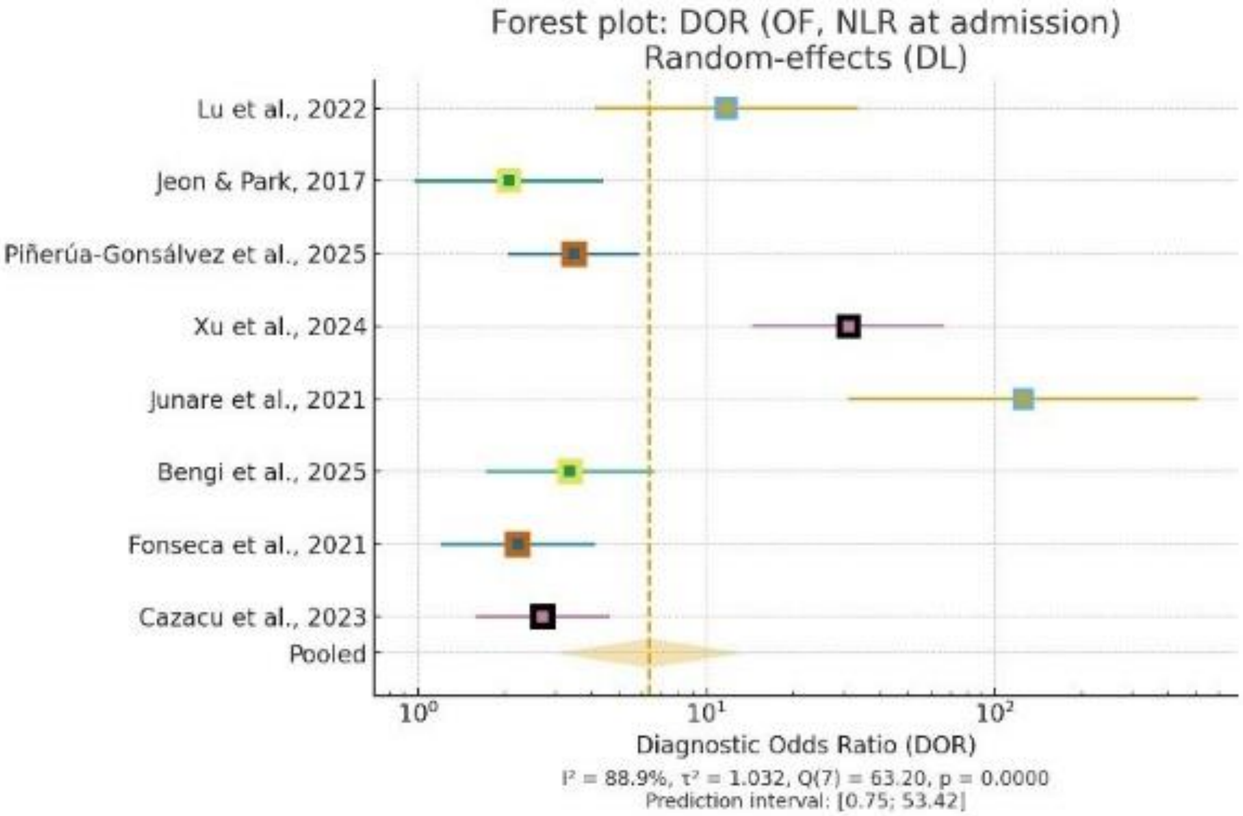

Supplementary Figure 34. Forest plot of log(DOR) for NLR at admission (POF). Random-effects model; individual study estimates with 95% CIs, the pooled estimate (RE), and the 95% prediction interval are shown.

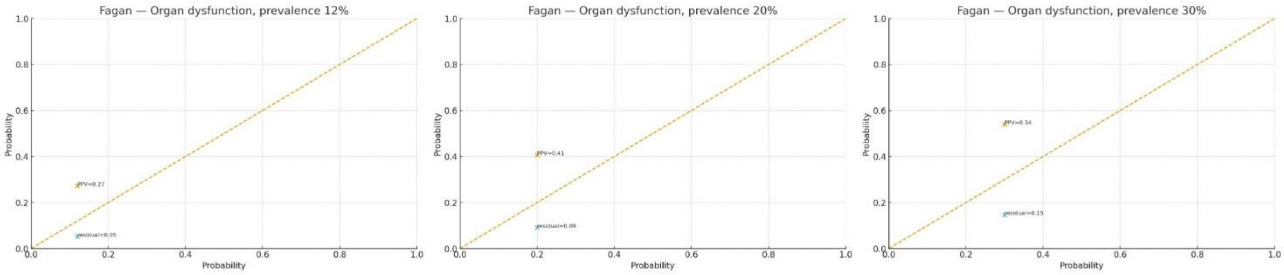

Supplementary Figure 35. Fagan plots for organ dysfunction using NLR at admission at prevalences of 12%, 20%, and 30%.

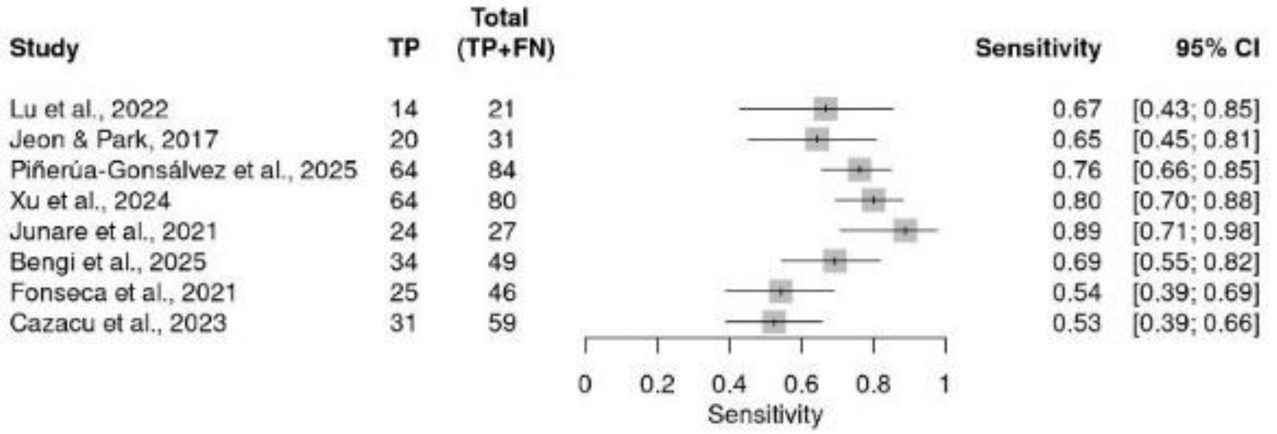

Supplementary Figure 36. Sensitivity of NLR for predicting infected pancreatic necrosis across the included studies.

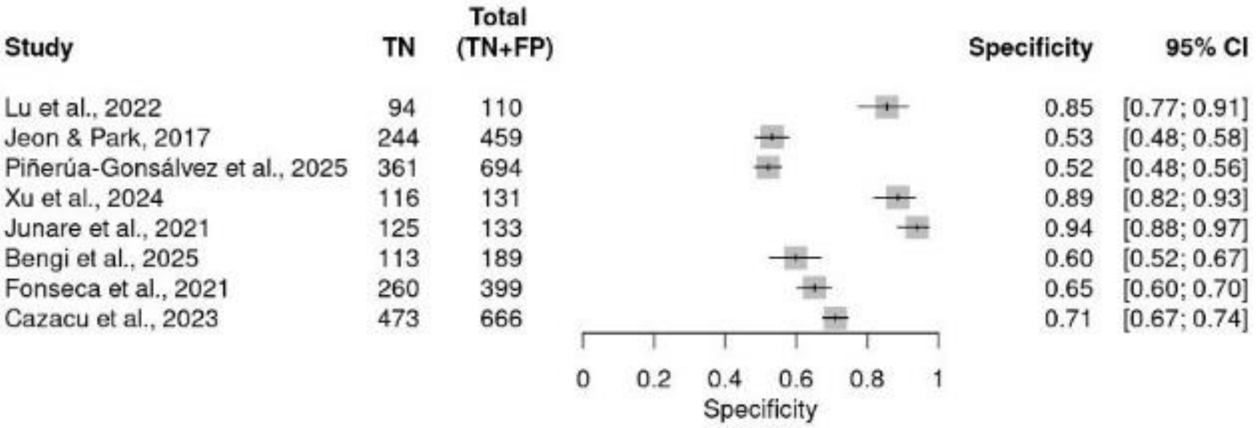

Supplementary Figure 37. Specificity of NLR for predicting infected pancreatic necrosis across the included studies.

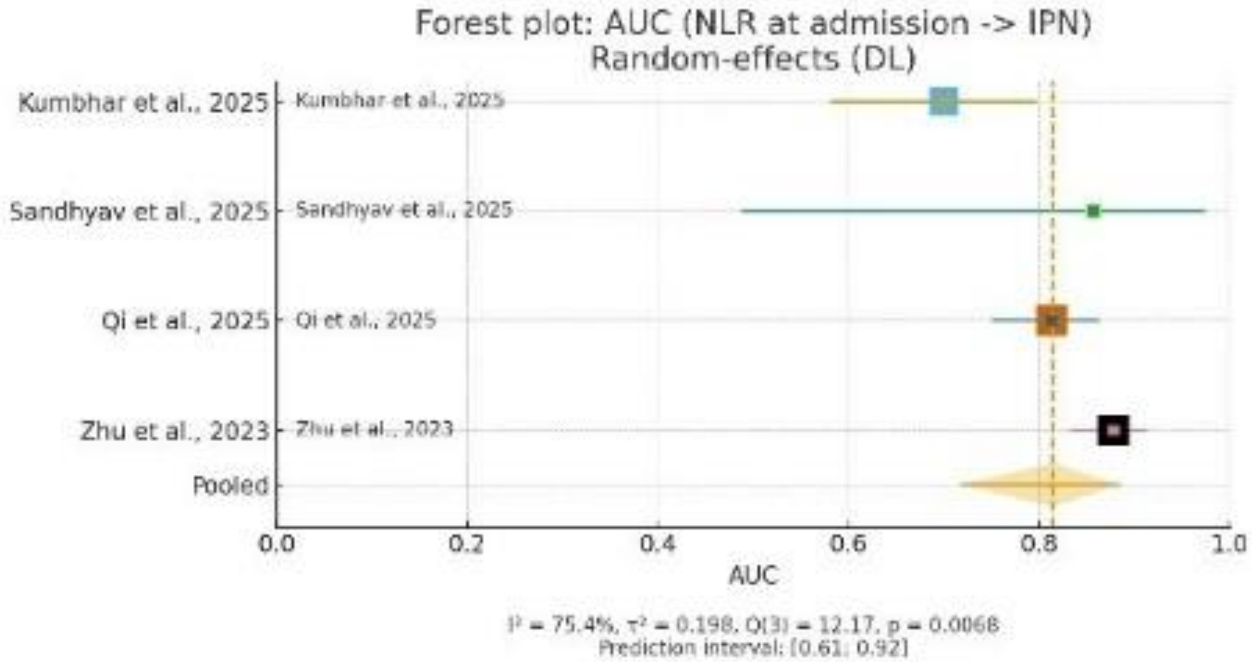

Supplementary Figure 38. Forest plot of AUC for NLR (IPN). Random-effects model; individual AUC values with 95% CIs and the pooled estimate (RE) with heterogeneity indicators (I^2, τ^2) are shown.

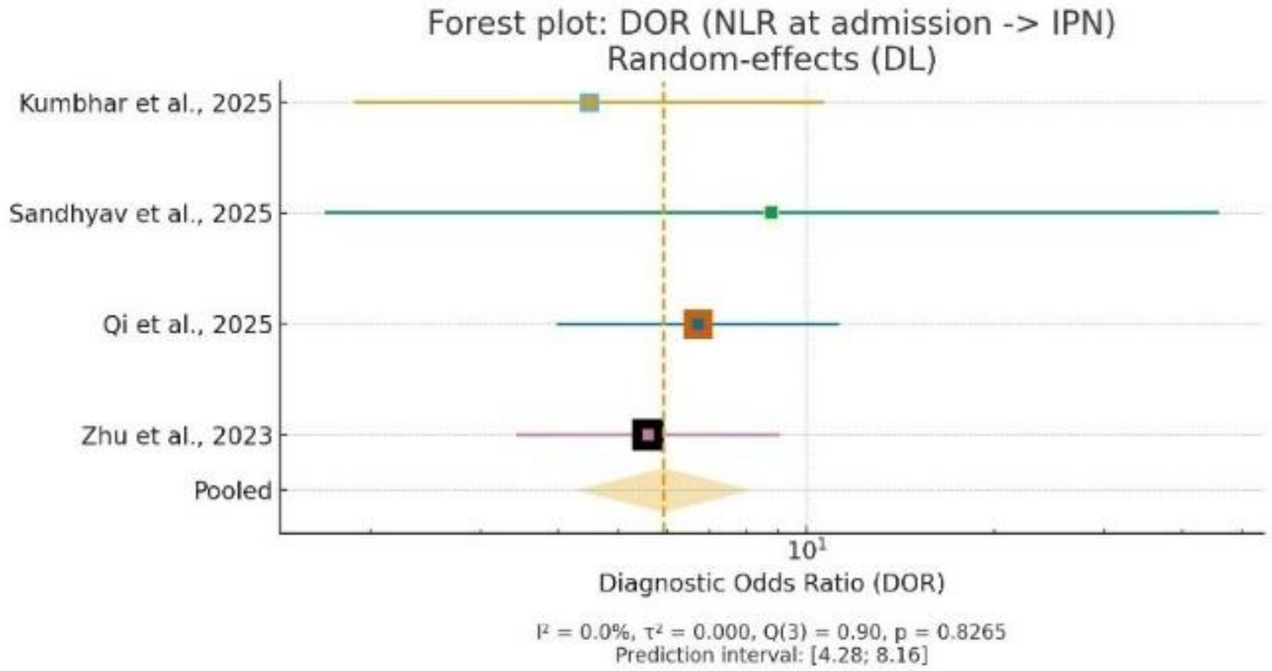

Supplementary Figure 39. Forest plot of log(DOR) for NLR (IPN). Random-effects model; individual study estimates with 95% CIs, the pooled estimate (RE), and the 95% prediction interval are shown.

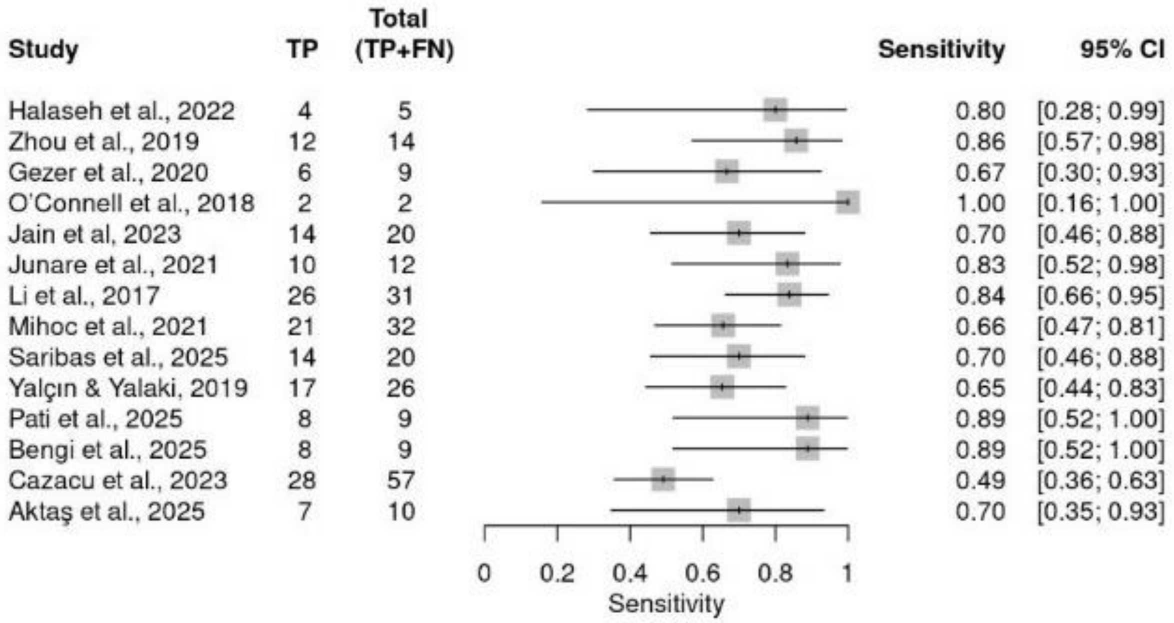

Supplementary Figure 40. Forest plot of admission NLR sensitivity for predicting mortality.

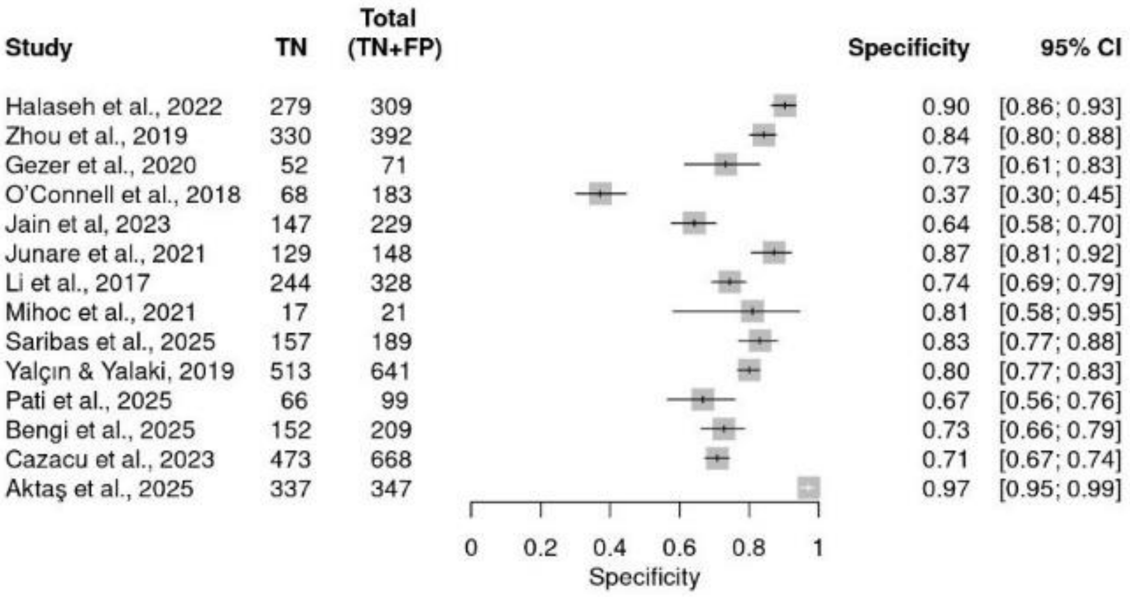

Supplementary Figure 41. Forest plot of admission NLR specificity for predicting mortality.

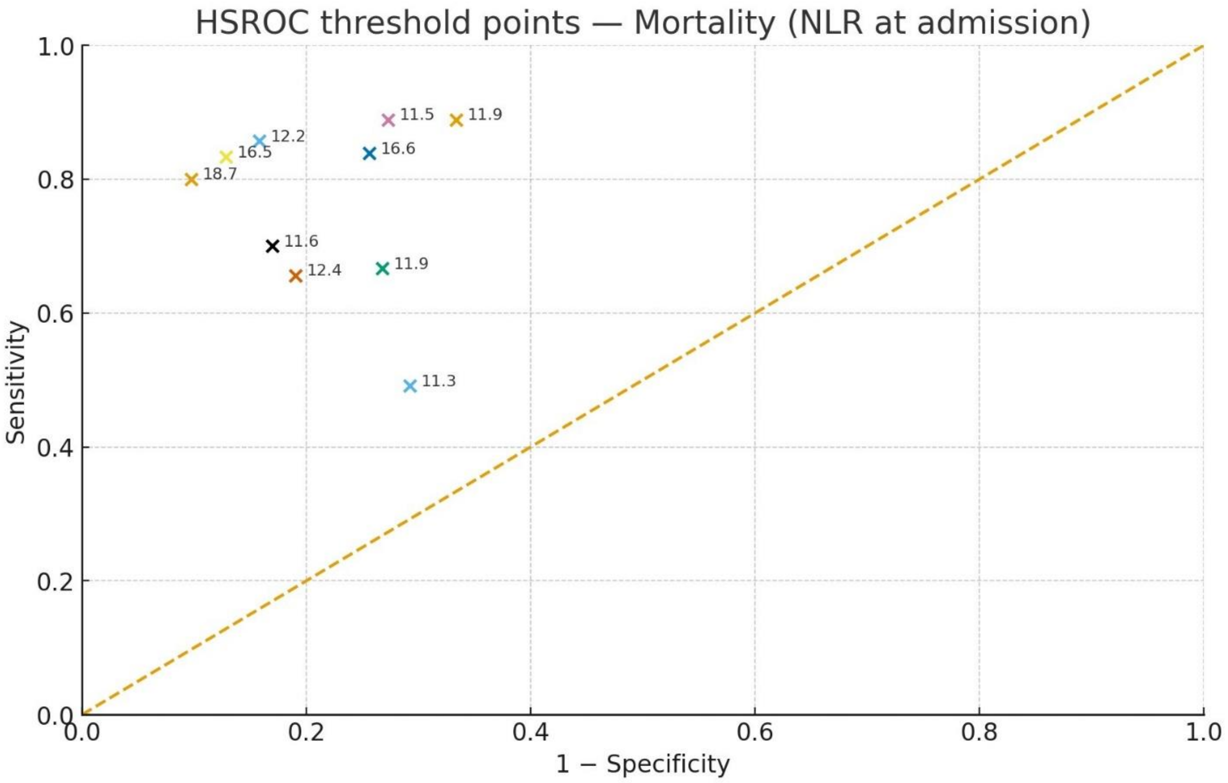

Supplementary Figure 42. HSROC threshold points for mortality prediction (NLR at admission).

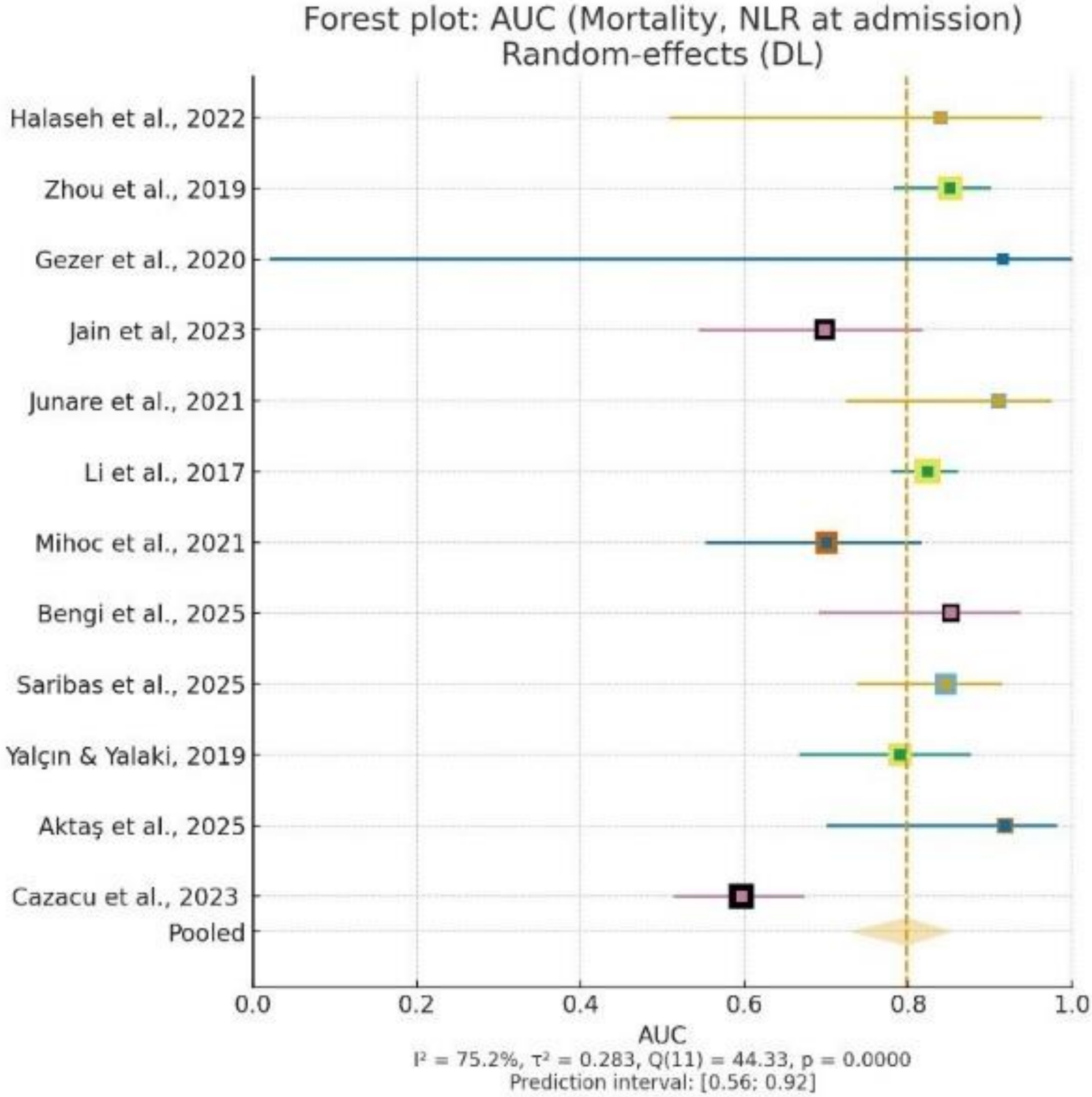

Supplementary Figure 43. Forest plot of AUC for admission NLR (mortality). Random-effects model; individual AUCs with 95% CIs and the pooled estimate (RE) with heterogeneity metrics (I^2, τ^2).

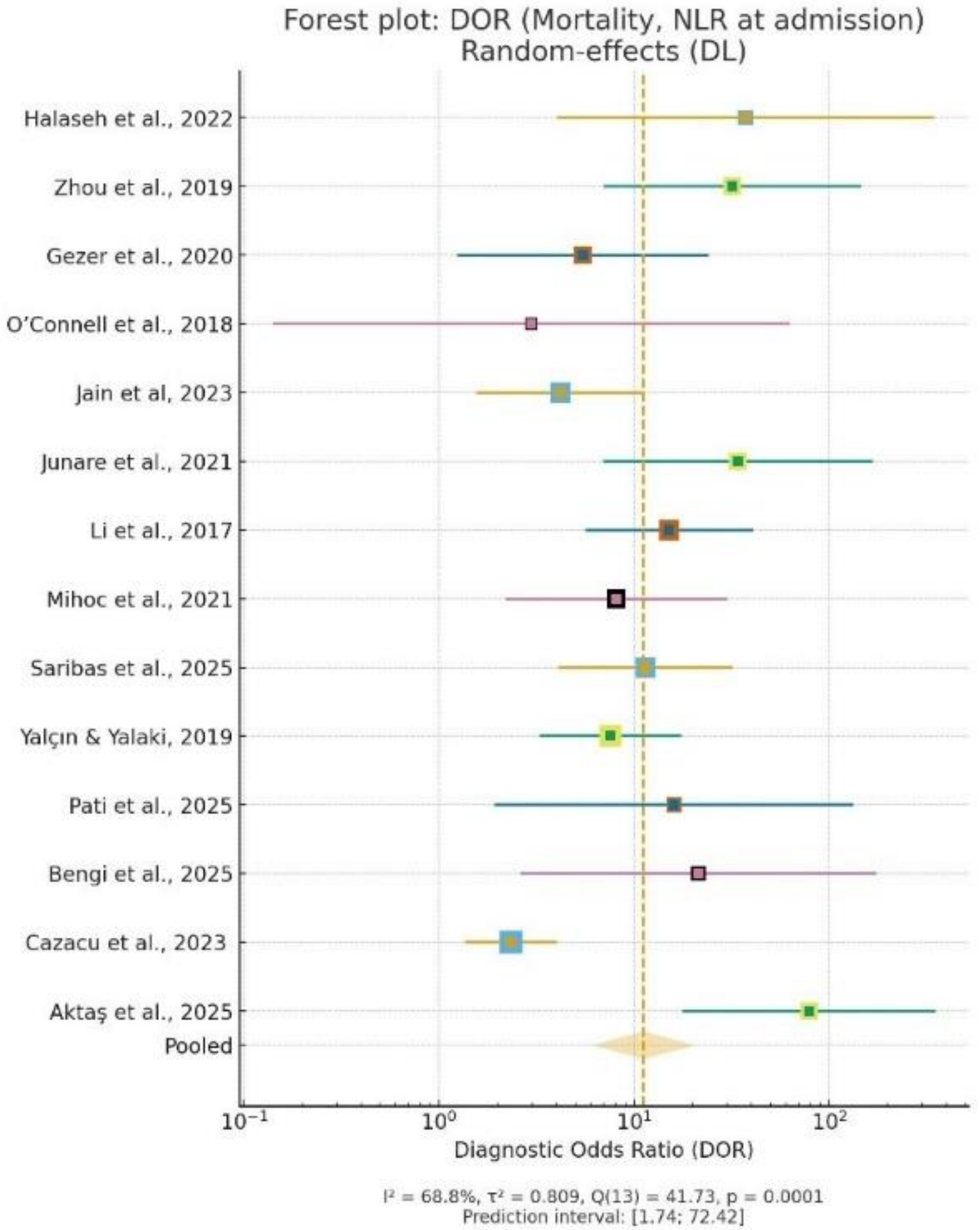

Supplementary Figure 44. Forest plot of log(OR) for admission NLR (mortality). Random-effects model; individual estimates with 95% CIs, the pooled estimate (RE), and the 95% prediction interval.

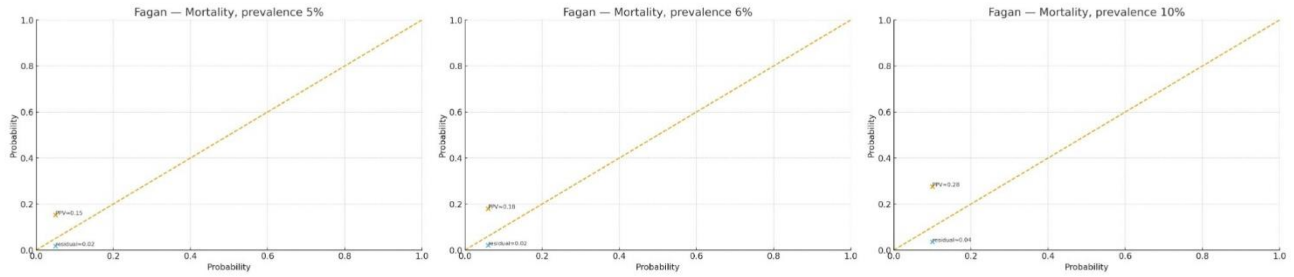

Supplementary Figure 45. Fagan nomograms for mortality prediction using admission NLR at prevalences of 5%, 6%, and 10%.
