# Supplementary material for: Neutrophil-to-lymphocyte ratio for primary risk stratification in acute pancreatitis: a systematic review and meta-analysis
Source: Front Med (Lausanne). 2026 Jan 13;12:1729339. doi: 10.3389/fmed.2025.1729339 (PMC12835348; doi:10.3389/fmed.2025.1729339)
Supplement: Supplementary file 2 [file Data_Sheet_2.pdf]

Supplementary Methods S1. Expanded statistical analysis (original text)

2.6 Statistical analysis

2.6.1 Prognostic factor (OR/RR associations)

• Effects (OR/RR) were pooled on the log(OR)/log(RR) scale using a random-effects model with τ2 estimated by REML; confidence intervals were constructed with the Hartung–Knapp–Sidik–Jonkman (HKSJ) approach.

• Between-study heterogeneity was assessed with I2, τ2, and the Q statistic (p-value); when ≥3 comparable studies were available, a 95% prediction interval (PI) was reported.

• Small-study effects/publication bias for OR/RR were evaluated with Egger's test, acknowledging its applicability to prognostic-factor meta-analyses; results were interpreted in the context of heterogeneity.

• If a single study reported multiple strata (time/threshold), the primary synthesis used a prespecified window (0/24/48 hours), prioritizing adjusted estimates; alternative strata were examined in sensitivity analyses.

• For zero cells in 2×2 tables (in sub-analyses requiring OR/RR computation), a standard continuity correction (0.5) was applied in sensitivity checks.

2.6.2 Prognostic accuracy (DTA approach)

• For each study with 2×2 tables, we calculated sensitivity/specificity (Se/Sp), LR±, and the diagnostic odds ratio (DOR); PPV/NPV were derived from pooled Se/Sp under prespecified prevalence scenarios (typically 10%/20%/30% and/or values characteristic of a given subgroup).

• The primary model was the bivariate HSROC (Reitsma) model, reporting pooled Se/Sp, LR±, DOR, bivariate I2, confidence/prediction ellipses, and, where appropriate, the median odds ratio (MOR) to describe between-study variability in Se and Sp.

• A threshold effect was assessed by the Spearman correlation between logit(Se) and logit(1–Sp). For visualization, HSROC curves were plotted with threshold-specific points (the cut-off annotated at each point) and/or curves stratified by threshold categories.

• Publication bias in DTA was assessed with Deeks' funnel asymmetry test (recommended for DTA): regression of ln(DOR) on 1/√n; a statistically significant non-zero intercept indicated possible bias. We prespecified that conclusions on publication bias for DTA would be based on Deeks' test, whereas Egger's test was applied only for OR/RR.

• AUC values were pooled in a random-effects model using inverse-variance weighting (weights = 1/SE2), with SE reconstructed from published 95% CIs; results were compared with the HSROC findings.

• For log(DOR), we conducted a separate random-effects meta-analysis (DerSimonian–Laird), reporting τ2 and the 95% PI (visualized with a forest plot).

• When the same threshold recurred in ≥2 independent studies, we performed threshold-specific aggregations (median Se/Sp, Youden's J index, pooled DOR on the log scale).

• Clinical calibration: Fagan nomograms were constructed for realistic prevalences (generally 10–30% or subgroup-specific values), and PPV/NPV were provided in the appendices (S-series tables/figures).

2.6.3 Thresholds (cut-offs) and their distribution

• For each sub-analysis (overall cohort; day 0/day 1/day 2; etiologic/clinical subgroups), published cut-offs were collected and their distribution summarized as the median, IQR, min–max, weighted median (by N), geometric mean, and, where appropriate, the 95% prediction interval for a new study on the log scale.

• The operational threshold for clinical application was selected by integrating: (i) the HSROC summary point (Se/Sp), (ii) the Youden's J and balanced-accuracy profiles, (iii) the distribution of published cut-offs, and (iv) the expected prevalence in the target population (impact on PPV/NPV).

2.6.4 Meta-regression and subgroups

• Planned meta-regressions/moderators (where ≥10 studies per analysis were available) included etiology (biliary/hypertriglyceridemic/other), threshold category, design (prospective/retrospective), continent/region, sampling time (0/24/48 hours), risk of bias (QUIPS/QUADAS-2), and enrollment period. For DTA, meta-regression was implemented within the bivariate HSROC framework by moderating logit(Se/Sp).

• Etiology-specific subgroups (biliary, hypertriglyceridemic) were pooled separately for severe AP; aggregation was performed when ≥2 studies with comparable thresholds/scales were available.

2.6.5 Sensitivity and auxiliary analyses

• Influential observations and estimate stability were examined using influence diagnostics, leave-one-out analyses, comparisons of REML- vs DL-based τ2, and alternative zero-cell corrections.

• Cross-checks ensured numerical reproducibility across the text and appendices: HSROC ↔ AUC ↔ log(DOR) ↔ thresholds/Youden ↔ Fagan/PPV/NPV.

2.6.6 Software and reproducibility

All statistical analyses were performed in R (version 4.5.1; R Core Team). For meta-analyses of effects (OR/RR), we used the metafor package (functions rma.uni, predict; influence diagnostics via influence.rma; stability via leave1out; and funnel-asymmetry testing via regtest for Egger). Where needed, the meta package (metagen, forest plotting) was employed to produce standardized forest plots. Prognostic-accuracy analyses were conducted with mada (madauni, reitsma, sroc); publication bias in DTA was evaluated with Deeks' test (regression of ln[DOR] on 1/√n). For AUC, we applied a random-effects model with inverse-variance weighting (weights = 1/SE2), reconstructing SE from published 95% CIs in base R. As an auxiliary check for DOR coherence and for rapid SROC construction in selected sub-analyses, we used Meta-DiSc 2.0. Data extraction and technical quality control were performed in Microsoft Excel with subsequent import into R. Reproducibility was ensured via standardized R scripts (fixed random seed using set.seed()), session protocol via sessionInfo(), and pinned package versions.
