# Supplementary material for: Neutrophil-to-lymphocyte ratio for primary risk stratification in acute pancreatitis: a systematic review and meta-analysis
Source: Front Med (Lausanne). 2026 Jan 13;12:1729339. doi: 10.3389/fmed.2025.1729339 (PMC12835348; doi:10.3389/fmed.2025.1729339)
Supplement: Supplementary file 3 [file Data_Sheet_3.pdf]

Supplementary Table 1. Heterogeneity and prediction interval (NLR on admission).

Model/component	Value
Random-effects (HK) OR — I^2, %	94.5
Random-effects (HK) OR — τ^2	1.3884
Random-effects (HK) OR — Prediction Interval (low–high)	0.41 – 49.75
Bivariate DTA — Var[logit(Se)]	0.353
Bivariate DTA — Var[logit(Sp)]	0.707
Bivariate DTA — I^2	0.793
Between-study median-OR (Se)	1.763
Between-study median-OR (Sp)	2.23

Supplementary Table 2. Published thresholds (cut-offs) for NLR on admission (day 0).

Weighted median	Unweighted median	IQR (low–high)	Minimum	Maximum	Geometric mean	95% PI for a new study (low–high)
9.21	9.0	7.40 – 10.34	2.43	14.70	8.06	3.65 – 17.79

Supplementary Table 3. PPV/NPV for NLR = 9 using bivariate LR± (Reitsma) (NLR on admission).

Prevalence	LR+	LR−	Post-test (positive)	Post-test (negative)	PPV	NPV
10%	2.835	0.307	0.24	0.03	0.24	0.97
20%	2.835	0.307	0.41	0.07	0.41	0.93
30%	2.835	0.307	0.55	0.12	0.55	0.88

Supplementary Table 4. Published thresholds (cut-offs) for NLR on day 1 (distribution summary).

n with cutoff	Weighted median	Median	IQR low	IQR high	Min	Max	Geometric mean	95% PI low	95% PI high
5	8.1	8.1	8.0	10.5	6.66	13.33	9.04	5.32	15.38

Supplementary Table 5. PPV/NPV and post-test probabilities for NLR = 8.1 at different prevalences (using LR+ = 2.16, LR− = 0.417) (day 1).

Prevalence	LR+	LR−	Post-test (positive)	Post-test (negative)	PPV	NPV
10%	2.16	0.417	0.19	0.04	0.19	0.96
20%	2.16	0.417	0.35	0.09	0.35	0.91
30%	2.16	0.417	0.48	0.15	0.48	0.85

Supplementary Table 6. Published thresholds (cut-offs) for NLR on day 2 (distribution summary).

n with cutoff	Weighted median	Median	IQR low	IQR high	Min	Max	Geometric mean
6	6.5	7.0	6.24	9.75	4.80	13.33	7.66

Supplementary Table 7. PPV/NPV and post-test probabilities for NLR ≈ 7 (day 2) at different prevalences (using LR+ = 2.380, LR− = 0.259).

Prevalence	LR+	LR−	Post-test (positive)	Post-test (negative)	PPV	NPV
10%	2.380	0.259	0.21	0.03	0.21	0.97
20%	2.380	0.259	0.37	0.06	0.37	0.94
27%	2.380	0.259	0.47	0.09	0.47	0.91
30%	2.380	0.259	0.50	0.10	0.50	0.90

Supplementary Table 8. Published thresholds (cut-offs) for NLR at admission – Organ dysfunction.

n with cutoff	Weighted median	Median	IQR low	IQR high	Min	Max	Geometric mean
8	8.41	8.80	7.49	12.15	5.03	16.30	9.26

Supplementary Table 9. PPV/NPV at different prevalences using bivariate LR± (LR+ = 2.74, LR− = 0.40) – Organ dysfunction.

Prevalence	LR+	LR−	PPV	NPV
12%	2.74	0.40	0.27	0.95
20%	2.74	0.40	0.41	0.91
30%	2.74	0.40	0.54	0.85

Supplementary Table 10. Mortality (NLR at admission): PPV/NPV scenarios using HSROC LR±

Prevalence	LR+	LR−	Post-test (positive)	Post-test (negative)	PPV	NPV
5%	3.44	0.34	0.15	0.02	0.15	0.98
6%	3.44	0.34	0.18	0.02	0.18	0.98
10%	3.44	0.34	0.28	0.04	0.28	0.96

Supplementary Table 11. Head-to-head comparisons of NLR with BISAP and APACHE II in acute pancreatitis (selected studies reporting AUC within the same cohort and time window)

**Study**	**Outcome / time window**	**AUC NLR**	**AUC BISAP**	**AUC APACHE II**	**Key note**
Zhou et al. [35]	Severe AP / on admission<br>Mortality / on admission	0.722<br>0.851	0.841<br>0.929	0.752<br>0.867	BISAP highest AUC for severity and mortality
Gezer et al. [37]	Severe AP / on admission<br>Mortality /on admission	0.726<br>0.915	0.836<br>0.847	—	BISAP better for severity; NLR higher AUC for mortality
Junare et al. [51]	Organ failure/on admission	0.940	0.932	0.845	NLR slightly highest for organ failure
Dancu et al. [53]	Severe AP / on admission<br>Severe AP / at 48 h	0.68<br>0.83	0.77<br>0.77	—	At admission BISAP > NLR; at 48 h NLR48h > BISAP
Kurtipek et al. [71]	Severe AP / on admission	0.763	0.867	0.893	Both BISAP and APACHE II outperformed NLR
Aktaş et al. [88]	Severe AP / at 48 h<br>Mortality / at 48 h	0.893<br>0.918	0.884<br>0.829	0.867<br>0.792	At 48 h NLR showed highest AUC among these three for both outcomes
